# Huntingtin Is Required for Epithelial Polarity through RAB11A-Mediated Apical Trafficking of PAR3-aPKC

**DOI:** 10.1371/journal.pbio.1002142

**Published:** 2015-05-05

**Authors:** Salah Elias, John Russel McGuire, Hua Yu, Sandrine Humbert

**Affiliations:** 1 Institut Curie, Orsay, France; 2 CNRS UMR 3306, Orsay, France; 3 INSERM U1005, Orsay, France; 4 Grenoble Institut des Neurosciences, University Grenoble Alpes, Grenoble, France; 5 INSERM U836, Grenoble, France; UT Southwestern Medical Center, UNITED STATES

## Abstract

The establishment of apical-basolateral polarity is important for both normal development and disease, for example, during tumorigenesis and metastasis. During this process, polarity complexes are targeted to the apical surface by a RAB11A-dependent mechanism. Huntingtin (HTT), the protein that is mutated in Huntington disease, acts as a scaffold for molecular motors and promotes microtubule-based dynamics. Here, we investigated the role of HTT in apical polarity during the morphogenesis of the mouse mammary epithelium. We found that the depletion of HTT from luminal cells in vivo alters mouse ductal morphogenesis and lumen formation. HTT is required for the apical localization of PAR3-aPKC during epithelial morphogenesis in virgin, pregnant, and lactating mice. We show that HTT forms a complex with PAR3, aPKC, and RAB11A and ensures the microtubule-dependent apical vesicular translocation of PAR3-aPKC through RAB11A. We thus propose that HTT regulates polarized vesicular transport, lumen formation and mammary epithelial morphogenesis.

## Abstract 2

Huntingtin—the protein that is aberrant in Huntington Disease—regulates apical vesicular trafficking to help establish apical-basolateral polarity during the development of mammary epithelia.

## Author Summary

In the adult mammary gland, tissue architecture is maintained through the regulation of the polarity of epithelial cells, which organize around a central cavity called the lumen. The mammary epithelium comprises a basal layer, which contains myoepithelial contractile cells and so-called mammary stem cells, and a luminal layer of cells organized around the lumen. The establishment of apical-basolateral polarity in luminal cells allows the separation of the apical and basolateral membranes and the maturation of cell–cell junctions. The protein complex composed of PAR3, PAR6, and aPKC regulates apical polarity in several tissues, including the mammary epithelium, and it is known that the loss of PAR3 and aPKC interferes with mammary gland development and promotes mammary tumor metastasis. RAB11A, a protein that regulates intracellular trafficking, coordinates apical translocation of PAR3-PAR6-aPKC. Huntingtin (HTT), the protein mutated in Huntington disease, modulates RAB11A activity and also regulates the microtubule-based vesicular trafficking in neurons. Using MCF10A, MDCK 2-D and 3-D cell cultures, and mouse models, we demonstrate here that HTT coordinates the apical vesicular trafficking of PAR3-PAR6-aPKC through RAB11A. We show that loss of HTT in luminal cells alters apical polarity, tissue architecture and the maturation of luminal cells during pregnancy and lactation in the mouse. Together, these findings uncover HTT-mediated vesicular trafficking as a new pathway in the establishment of epithelial apical polarity, with potential implications for health and disease.

## Introduction

Epithelial cells in glandular and tubular epithelial systems are organized as one layer surrounding a lumen. The establishment of apical-basolateral polarity in these systems is characterized by the formation of cell–cell adherens and tight junctions and accompanies lumen formation (reviewed in [1–3]). This organization provides a functional barrier that regulates the polarized secretion and intake of molecules. Cell polarity complexes, which were originally identified in model organisms such as yeast, worms, and flies, are highly evolutionarily conserved [4]. Three major polarity complexes have been identified. The PAR polarity complex, which includes Partitioning Defective 3 and 6 (PAR3 and PAR6), atypical protein kinase C (aPKC) and cell division control protein 42 (CDC42) proteins, promotes the establishment of the apical-basal membrane border. The Crumbs (CRB) complex, which is required to establish the apical membrane, is composed of the transmembrane protein CRB and the associated cytoplasmic proteins, PALS1 (also known as MPP5) and PALS1-associated tight junction protein (PATJ; also known as INADL). Finally, the Scribble complex, which is composed of scribble homolog (SCRIB), lethal giant larvae homolog (LGL; also known as LLGL), and disc-large homolog (DLG) proteins, defines the basolateral plasma domain. In *Drosophila*, these complexes interact and establish the apical and basolateral surfaces of epithelial cells by a system of mutual exclusion [5,6].

The PAR complex is a master regulator of polarity and is involved in polarity and spatial organization in almost all metazoan cells [7]. Mammalian PAR3 is localized to tight junctions at the apical/lateral boundary [8], and functions in their assembly [9], whereas PAR6 and aPKC maintain the integrity of the apical domain [10]. All of these proteins interact directly with each other. PAR6 acts as a targeting subunit for aPKC, and it recruits the CRB complex [11,12] and LGL as substrates [13]. The binding of PAR3 to PAR6, which forms a complex with aPKC, is required for the delivery of aPKC to the apical surface [14,15]. Moreover, the interaction of PAR3 with aPKC is essential for the restricted localization of these proteins to the apical region [15]. In the mammary gland, this interaction is essential for the regulation of progenitor differentiation and epithelial morphogenesis [15].

Formation of the apical surface, the first step of lumen morphogenesis, involves the coordination of the trafficking machinery and the polarity complexes. In mammalian cells, vesicles containing apical membrane components are delivered to a region named the apical membrane initiation site (AMIS) where the lumen begins [1,16]. This region is delineated by PAR3, aPKC, and the exocyst subunit SEC8. In polarized cells, trafficking from recycling endosomes is regulated by several members of the family of RAB GTPases. In particular, RAB11 controls vesicle trafficking in apical recycling endosomes and is necessary for epithelial morphogenesis [17,18]. Similarly, during lumen formation, the trafficking of vesicles containing apical membrane components depends on RAB11 [16]. The targeting of apical vesicles containing podocalyxin (PCX) to the AMIS is regulated by RAB11A together with RAB8 and RABIN8, a RAB8-specific GEF that is activated by RAB11A [16]. The PAR complex targets SEC8-SEC10 to the AMIS, and recruits SEC15A-RAB8A-RAB11A vesicles to generate the pre-apical patch (PAP) [16]. This mechanism leads to the localization of CDC42 to the apical membrane, where it activates the PAR complex. Although the core complexes involved in these mutually interdependent processes are well characterized, regulatory factors that couple polarity proteins to the membrane transport machinery have not been identified.

Huntingtin (HTT), the protein mutated in Huntington disease, acts as a molecular scaffold and promotes intracellular dynamics. HTT associates with vesicles and microtubules. It is crucial for vesicular trafficking and affects axonal transport and endocytosis. HTT binds dynein and HAP1 directly [19], and kinesin [20] and the dynactin subunit p150^*Glued*^ [21] indirectly. HTT facilitates the transport of several cargoes along microtubules [22–24]. HTT also mediates vesicle recycling during endocytosis by activating RAB11 [25]. These functions have consequences for a wide variety of cellular events mostly described in the nervous system during both development and the maintenance of homeostasis in adults. For instance, through its function as a regulator of microtubule-based dynamics, HTT influences the division of progenitors at the ventricular zone during cortical development [26], the maturation of newly generated neurons during adult hippocampal neurogenesis [27] and ciliogenesis in ependymal cells [28]. However, HTT expression is ubiquitous, and this raises questions concerning the functions of HTT in tissues outside the central nervous system. We previously showed that HTT is detectable in healthy mammary tissue and mammary tumors where it regulates tumor progression [29]. HTT is required in mammary basal progenitors for appropriate spindle orientation and for the determination of cell fate [30]. Here, we focused on the function of HTT in the establishment of apical polarity during the morphogenesis of the mouse mammary epithelium. We propose that HTT regulates apical vesicular transport, which enables the proper targeting of polarity proteins and the correct establishment of subsequent luminogenesis.

## Results

### The Depletion of Huntingtin from Luminal Cells In Vivo Alters Ductal Morphogenesis and Lumen Formation

**Fig 1:**
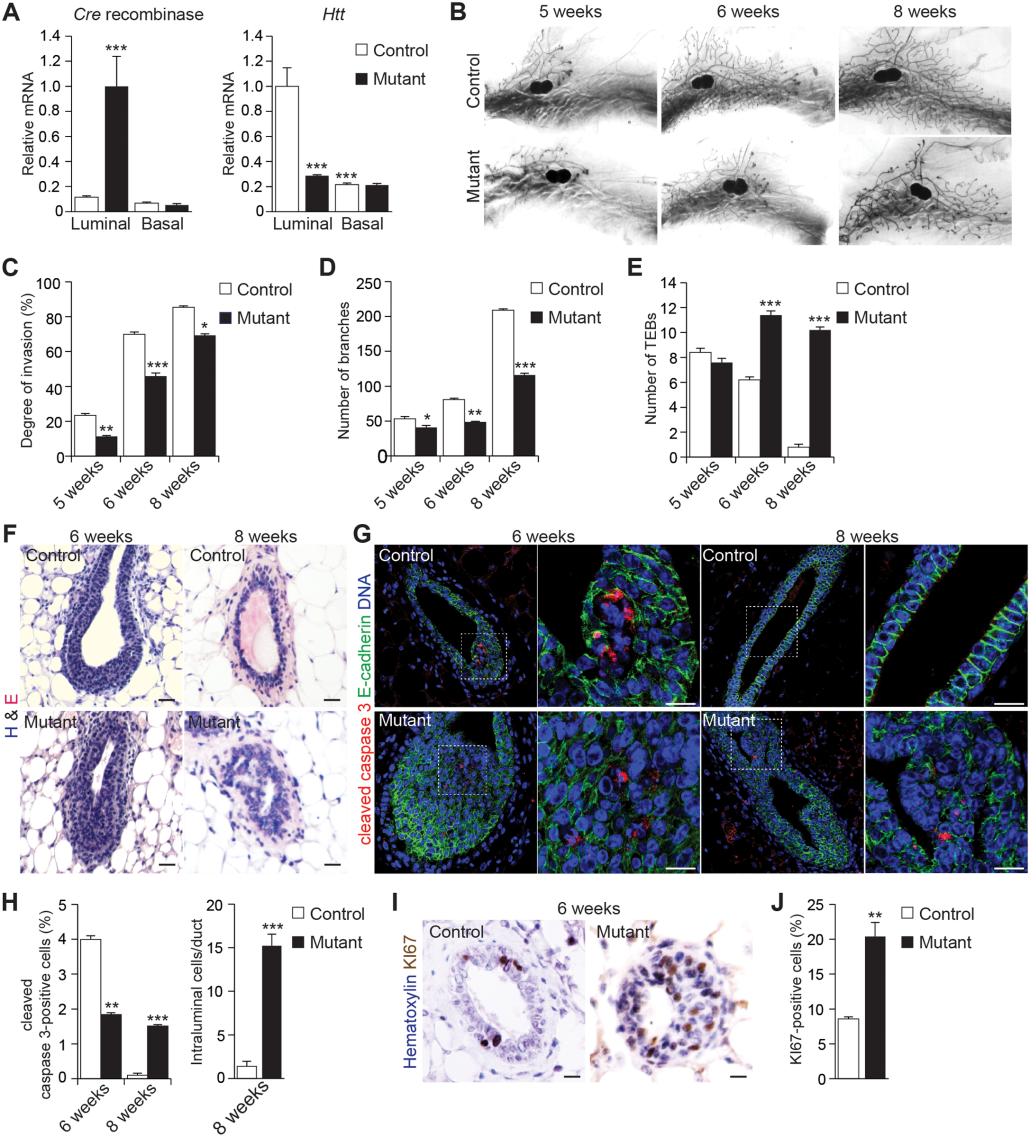
MMTV-driven loss of HTT affects ductal morphogenesis. (A) Quantitative real-time RT-PCR analysis of *Cre* and *Htt* gene in basal and luminal mammary epithelial cells from 16-wk-old virgin mice. Data are presented as means obtained in three independent experiments (control: five mice per experiment, mutant: five mice per experiment). (B) Carmine-stained whole mounts of mammary glands and hematoxylin and eosin (H&E) staining. (C) Degree of ductal invasion of the fat pad in virgin mammary glands. (D) Number of branches in virgin mammary glands. (E) Number of terminal end buds (TEBs) in virgin mammary glands. (F) H&E staining of mammary gland sections. (G) Mammary gland sections stained for E-cadherin and cleaved caspase 3. (H) Percentage of cleaved caspase 3-positive cells and number of intraluminal cells per duct. (I) Mammary gland sections stained for KI67. (J) Percentage of KI67-positive cells. Number of mice analyzed are the same in C-E, H, J: control: *n* = 5 mice; mutant: *n* = 7 mice. All scale bars, 10 μm. Error bars, standard error of the mean (SEM). *p<0.05; **p<0.01; ***p<0.001. Complete statistical analyses with number of measures are detailed in S1 Data.

We recently showed that depletion of HTT from the basal compartment in the mammary gland results in altered morphological and functional differentiation [30]. However, the abundance of HTT is higher in luminal cells (LCs) than in basal cells (BCs) (Fig 1A) [30]. We sought to address whether HTT expression specifically in LCs is essential for epithelial morphogenesis; therefore, we deleted HTT from the luminal cell layer of the mammary epithelium by crossing *Htt* ^*flox/flox*^ mice harboring floxed *Htt* alleles [31] with transgenic mice expressing *Cre* recombinase under the control of the mouse mammary tumor virus (MMTV) promoter [32]. *Cre* expression was mostly confined to the luminal cell population (Fig 1A). The abundance of *Htt* transcripts was 72% lower in LCs from MMTV*Cre*;*Htt* ^*flox/flox*^ (mutant) epithelium than in those from control epithelium, whereas mammary *Htt* transcript levels were similar in control and mutant BCs. Thus, HTT is specifically depleted in luminal cells in MMTV*Cre*;*Htt* ^*flox/flox*^ mice.

We then performed whole mount staining with fourth abdominal mammary glands isolated from mutant and control mice at the age of 5, 6, and 8 wk to measure ductal elongation and bifurcation. The direct visualization of ductal trees showed that ductal elongation and bifurcation were less extensive in mutant mice than in control mice (Fig 1B). We quantified these effects by measuring the percentage of the fat-pad area covered by the ductal structures and the number of branches; both were significantly lower in mutant mice than in control mice at all stages analyzed (Fig 1C and 1D). Interestingly, the number of terminal end buds (TEBs) in 6- and 8-wk-old glands (Fig 1E) was significantly higher in mutant mice than in control mice. At 12 wk, which marks the end of puberty in mice, ductal extension and branching were similar between mutant and control mice, and the effect of HTT deletion disappeared (S1A and S1B Fig). These findings suggest that loss of HTT delays ductal elongation and bifurcation in the mammary tree during puberty.

We performed hematoxylin and eosin staining on serial sections of mammary glands from 6- and 8-wk-old control and mutant mice (Fig 1F). Although control TEBs showed a well-defined lumen at 6 wk, the structures from mutant mice were partially filled with cells. At 8 wk, control ducts were completely hollow, whereas mutant ducts displayed an aberrant architecture and contained many intraluminal cells (Fig 1F). We hypothesized that these defects were linked to alterations in cell death and proliferation. Thus, we stained sections from control and mutant mammary ducts for cleaved caspase-3 to analyze apoptosis (Fig 1G). Globally there were a high number of intraluminal cells in mutant TEBs; however, within this population, the proportion of apoptotic cells was lower in mutant TEBs than control TEBs at 6 wk (1.84% ± 0.05% in mutant versus 3.99% ± 0.1% in control mice; Fig 1G and 1H). In contrast with controls, ducts from 8-wk-old mutant mice still displayed apoptotic cells and a high number of intraluminal cells (15.2% ± 1.36% in mutant versus 1.4% ± 0.57% in control mice; Fig 1G and 1H). Furthermore, KI67 immunostaining showed that the percentage of proliferating cells was higher in ducts from 6-wk-old mutant mice than in those from control mice of the same age (20.4% ± 2% in mutant versus 8.6% ± 0.57% in control mice; Fig 1I and 1J). At 8 wk, the percentage of proliferating cells was similar in control and mutant mice (S1C Fig).

These in vivo data suggest that HTT may result in delayed apoptotic-mediated clearing of intraluminal cells. To confirm this hypothesis, we used the human MCF-10A cells, which form acini in 3-D culture by 20 d of morphogenesis by luminal cells clearing through apoptosis-mediated anoikis [33]. HTT deletion using specific shRNA blocked apoptosis-mediated luminal clearing, resulting in malformed acini filled with cells (S2A–S2E Fig). The acini formed when HTT levels were lowered were significantly larger than in control condition (S2B, S2D and S2F Fig). Thus, the loss of HTT alters ductal morphogenesis and results in delayed intraluminal cell death and a malformed lumen.

**Fig 2:**
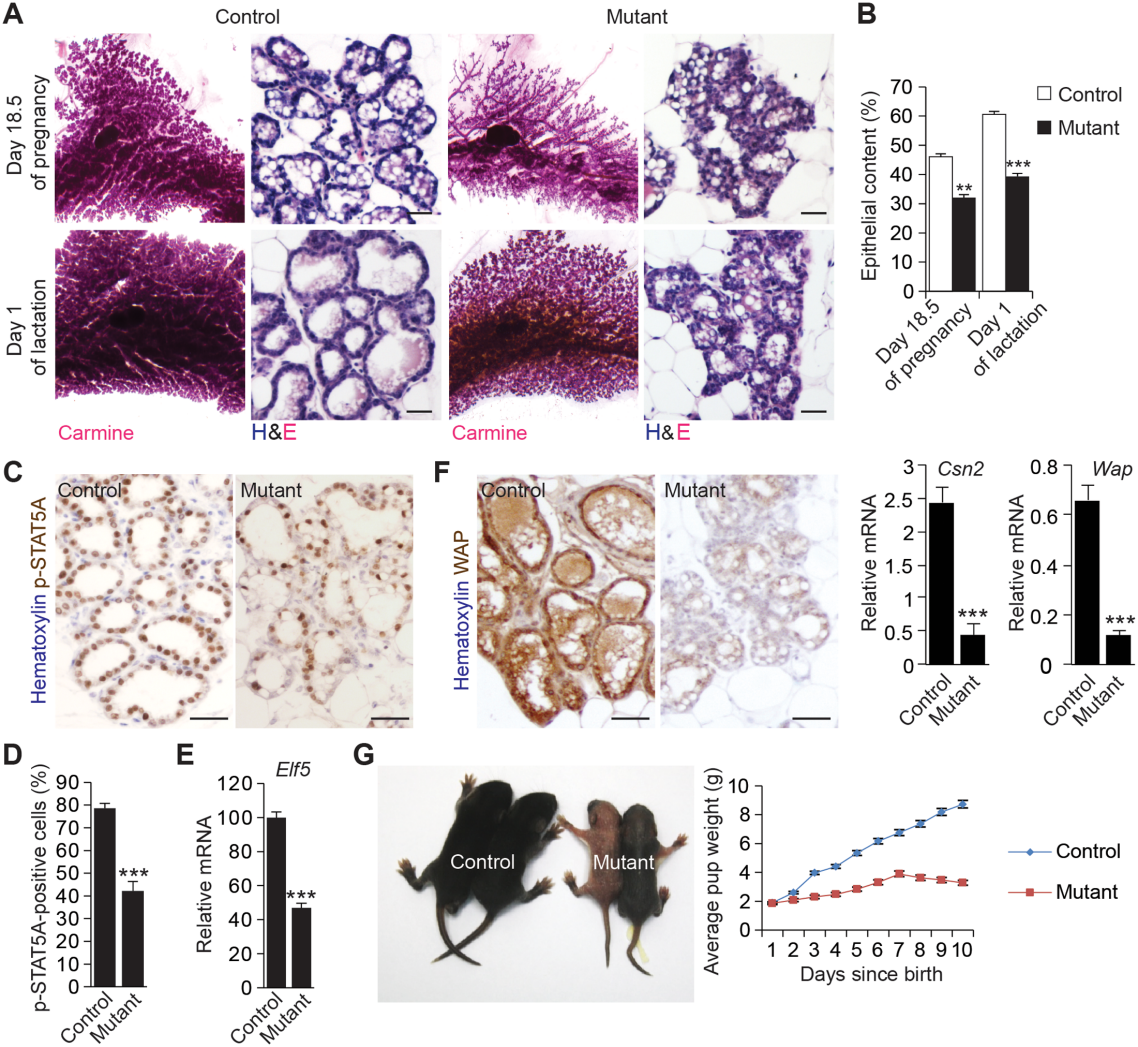
MMTV-driven loss of HTT affects alveolar morphogenesis. (A) Carmine-stained whole mounts of mammary glands and hematoxylin and eosin staining (H&E). Scale bars, 50 μm. (B) Quantification of the epithelial content (control: *n* = 6 mice; mutant: *n* = 6 mice). (C) Mammary gland sections stained for p-STAT5A. Scale bars, 10 μm. (D) Percentage of p-STAT5A-positive cells (control: *n* = 6 mice; mutant: *n* = 6 mice). (E) Quantitative real-time RT-PCR analysis of *Elf5* gene expression. Data are presented as means obtained in three independent experiments (control: three mice per experiment, mutant: three mice per experiment). (F) Mammary gland sections stained for WAP. Scale bars, 10 μm. The histograms show quantitative real-time RT-PCR analysis of *Csn2* and *Wap* gene expression. The values were normalized to *Krt8* expression. Data are presented as means obtained in three independent experiments (control: three mice per experiment, mutant: three mice per experiment). (G) Average weight of pups. Data are presented as means obtained in two independent experiments (control: three mice per experiment, mutant: three mice per experiment). Error bars, SEM. ** *p*<0.01; *** *p*<0.001. Complete statistical analyses with number of measures are detailed in S1 Data.

We also investigated how the loss of HTT in luminal cells affected the differentiation of the mammary gland at day 18.5 of pregnancy and day 1 of lactation. Both the number of secretory alveoli and the percentage of epithelial cells were lower in mutant glands than in control glands (Fig 2A and 2B). On day 18.5 of pregnancy and day 1 of lactation, there were fewer well-developed alveoli in mutant glands than in control glands. In controls, the large cytoplasmic lipid droplets in luminal alveolar cells on day 18.5 of pregnancy were replaced with small lipid droplets at the luminal surface on day 1 of lactation (Fig 2A). In mutant mammary glands, the large cytoplasmic droplets remained in the alveolar cells on day 1 of lactation. We investigated the functional consequences of these epithelial defects by analyzing the subcellular location of signal transducer and activator of transcription 5A (STAT5A) on day 1 of lactation (Fig 2C and 2D). The abundance of phosphorylated STAT5A in the nucleus (the active form of STAT5A) was lower in mutant alveolar cells than in control glands. The abundance of transcripts encoding the transcription factor ELF5 (*Elf5*), which is crucial for lobuloalveolar morphogenesis [34], was significantly lower in mutant alveoli than in control alveoli (Fig 2E). Consistent with these observations, immunolabeling showed that the abundance of the milk whey acid protein (WAP) was lower in mutant glands than in control glands, and the RT-PCR revealed that the same was true for RNAs encoding the milk proteins β-casein (*Csn2*) and WAP (*Wap*) (Fig 2F). Ultimately, mutant mice failed to nurse their pups, which displayed severe weight defects (Fig 2G).

Overall, these findings show that the loss of HTT in LCs alters lumen formation, ductal morphogenesis, and tissue architecture at different stages of mammary gland development and has functional consequences during lactation.

### Huntingtin Is Required for the Apical Localization of PAR3-aPKC during Epithelial Morphogenesis in Virgin, Pregnant, and Lactating Mice

**Fig 3:**
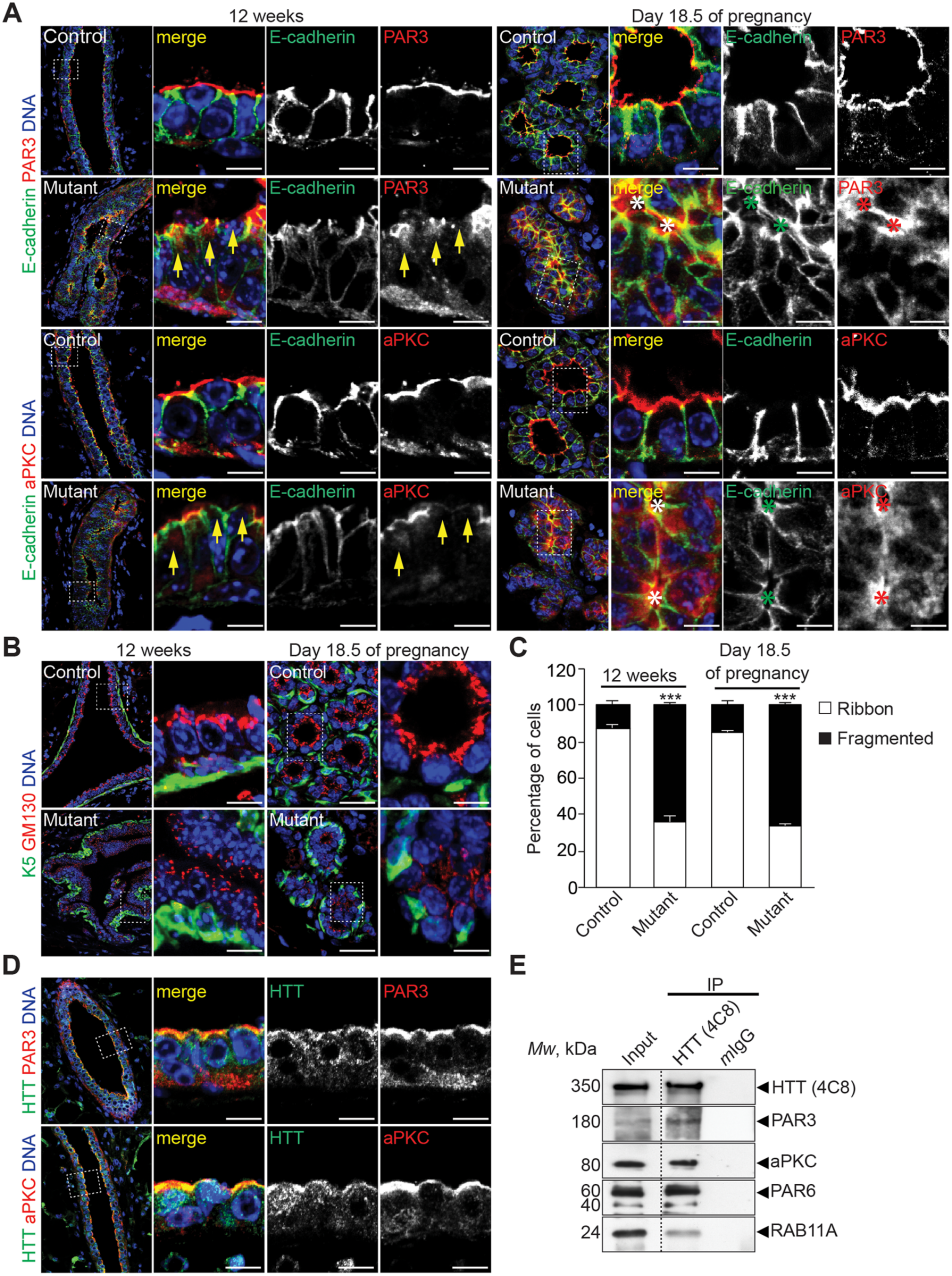
HTT is required for apical localization of PAR3 and aPKC in vivo. (A) Mammary gland sections stained for E-cadherin and PAR3 or aPKC. Arrows indicate cytoplasmic accumulation and impaired localization of PAR3 and aPKC to the apical surface; asterisks indicate small lumens. (B) Mammary gland sections stained for keratin 5 (K5) and GM130. (C) Percentage of LCs showing ribbon-like and fragmented localization of GM130 (control: *n* = 4 mice; mutant: *n* = 4 mice). (D) Mammary gland sections stained for HTT 4C8 and PAR3 or aPKC. (E) HTT/PAR3/PAR6/aPKC/RAB11A complexes were immunoprecipitated from MCF-10A cells. Mouse IgG (mIgG) was used as a negative control. The immunoprecipitates (IP) were analyzed by western blotting. All scale bars, 10 μm. Error bars, SEM. *** *p*<0.001. Complete statistical analyses with number of measures are detailed in S1 Data.

We then analyzed how HTT deficiency in LCs affects epithelial cell polarity. We compared the localization of PAR3 and aPKC in LCs from 12-wk-old virgin mutant and control mice (Fig 3A). In control glands, PAR3 and aPKC were localized at tight junctions and the apical surface of LCs, whereas in mutant ducts, PAR3 and aPKC labeling was more diffuse, and both proteins accumulated in the cytoplasm. We also examined the localization of E-cadherin, a marker of adherens junctions (Fig 3A). As expected, E-cadherin was enriched at the lateral compartment in control LCs. By contrast, in mutant LCs, it accumulated abnormally with PAR3-aPKC at the apical surface and was also dispersed in the cytoplasm. This was associated with defects in epithelial architecture and lumen malformation. Apical localization of PAR3-aPKC was also altered when mutant epithelia formed lumens (Fig 3A; arrows). We also determined the distribution of PAR3, aPKC, and E-cadherin in LCs of control and mutant epithelia on day 18.5 of gestation and day 1 of lactation (Figs 3A and S3A). Consistent with the morphological defects observed at all stages (Figs 1 and 2), the defects caused by the loss of HTT in pregnant and lactating mice were similar to those seen in virgin mice and included the mislocalization of PAR3, aPKC, and E-cadherin and lumen malformation (see asterisks).

We also analyzed the Golgi distribution by immunostaining using an antibody directed against the Golgi matrix protein GM130 (Figs 3B and S3B). In control epithelia, the Golgi apparatus localization was polarized in an apical position facing the lumen in most of LCs of control epithelia at all stages analyzed (Figs 3B, 3C, S3B and S3C). In the majority of mutant LCs, however, we found that the Golgi apparatus was dispersed within the soma and did not show a characteristic polarized distribution. We confirmed these observations in 3-D cultures of MCF-10A (S3D–S3F Fig). While the Golgi apparatus was dispersed in the absence of HTT, it still displayed a perinuclear distribution. In agreement, the microtubule network, which maintains the Golgi apparatus in the perinuclear area [35], was comparable in control and shHTT-treated MCF-10A cells (S4 Fig). Thus, the absence of HTT in luminal cells alters their polarization.

We then asked whether HTT directly regulates the polarity complex. We determined whether HTT colocalizes with PAR3 and aPKC in mammary glands from 12-wk-old control mice (Fig 3D). HTT colocalized with PAR3 and aPKC at the apical surface of LCs. In particular, HTT was enriched at tight junctions. Furthermore, PAR3 and aPKC coimmunoprecipitated with HTT in extracts of mammary epithelial MCF-10A cells (Fig 3E). Consistent with these data, affinity-purification mass spectrometry previously showed that PAR3 and aPKC form a complex with HTT in cortical neurons [36]. Although PAR6 has not been reported to interact with HTT, it belongs to the PAR polarity complex [7] and also coimmunoprecipitated with HTT, PAR3, and aPKC. Thus, HTT is associated with components of the PAR polarity complex and may regulate epithelial polarity through this interaction.

### Huntingtin Localizes with PAR3-aPKC at Cytoplasmic Vesicles and Modulates Their Apical Translocation in 3-D Culture

**Fig 4:**
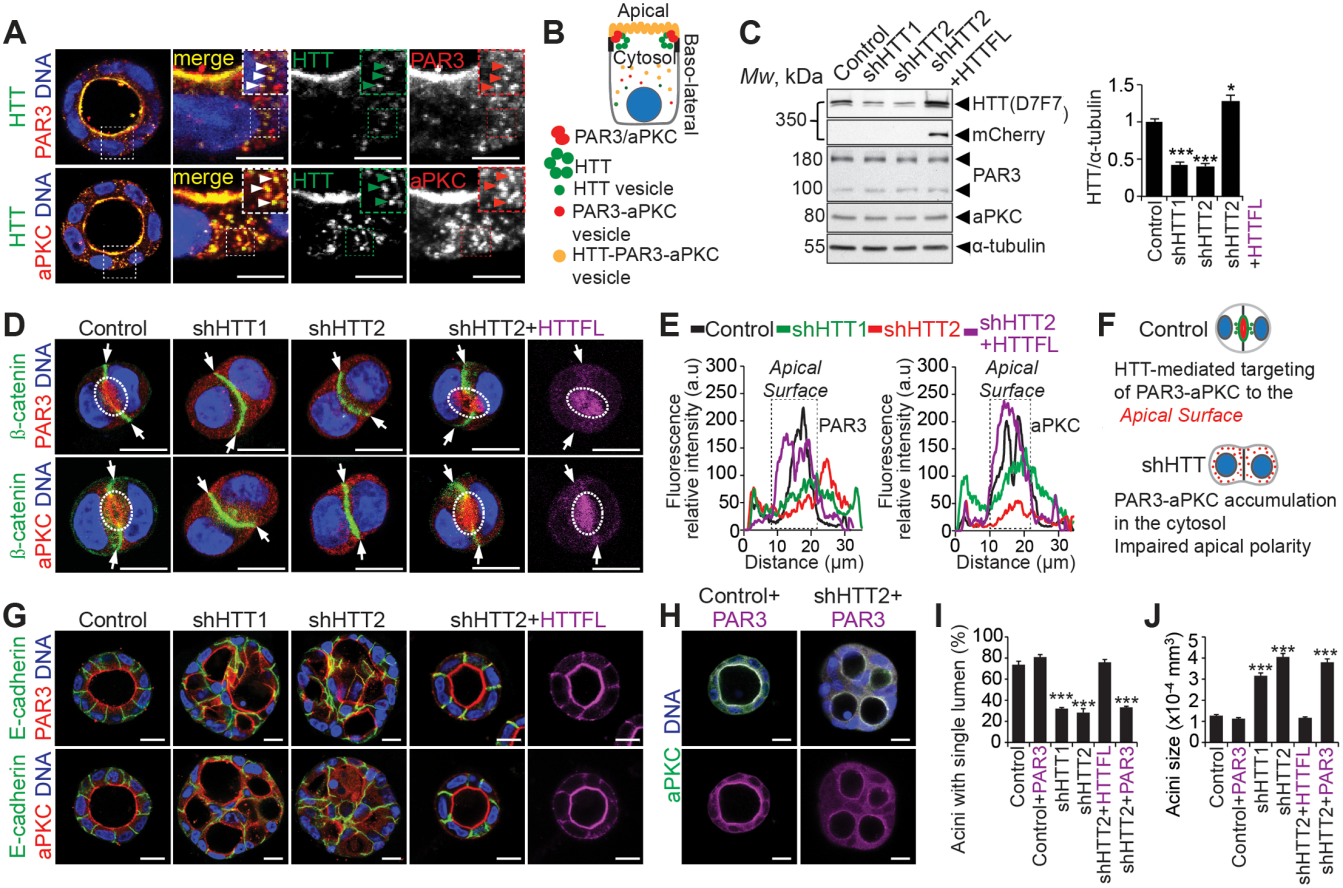
HTT regulates apical vesicular trafficking of PAR3-aPKC during cystogenesis. (A) Four-day MDCK 3-D cultures stained for HTT 4C8 and PAR3 or aPKC. Arrowheads indicate localization of HTT and PAR3 or aPKC to vesicular-like structures. Colocalization of HTT and PAR3 or aPKC is displayed in yellow (merge). (B) Illustration showing the HTT/PAR3/PAR6/aPKC complex localization on apical membrane and vesicles. (C) Western blotting of MDCK cell extracts. The histogram corresponds to the quantification of HTT levels. (D) 24 h MDCK 3-D structures stained for ß-catenin and PAR3 or aPKC. HTTFL is tagged with mCherry and staining is displayed in magenta. Arrows indicate the basolateral compartment and dashed ellipses indicate the apical surface. (E) Representative line-scan analysis (relative fluorescence intensity; at least 20 cells were analyzed per condition). (F) Illustration showing the role of HTT in PAR3-aPKC apical vesicular trafficking. (G) Four-day MDCK 3-D structures stained for E-cadherin and PAR3 or aPKC. (H) Four-day MDCK 3-D structures stained for aPKC. PAR3-GFP staining is displayed in magenta and the colocalization of aPKC and PAR3-GFP appears in white. (I) Percentage of acini with normal lumen. (J) Quantification of acini size. (I and J) Control: *n* = 125 acini, Control + PAR3: *n* = 102 acini, shHTT1: *n* = 149 acini, shHTT2: *n* = 114 acini, shHTT2 + HTT: *n* = 163 acini, shHTT2 + PAR3: *n* = 89 acini. All scale bars, 10 μm. Error bars, SEM. *** *p*<0.001. Complete statistical analyses with number of measures are detailed in S1 Data.

We then investigated the mechanisms by which HTT regulates apical polarity during epithelial morphogenesis. MCF-10A and primary mammary epithelial cells are useful to assess several aspects of mammary epithelial morphogenesis in 3-D culture [33] (S2 and S3D–S3F Figs). For instance, the localization of polarity markers such as GM130 can be assessed in MCF-10A cysts with already-formed lumen (S3D–S3F Fig). However, MCF-10A and primary cells form lumen by apoptotic hollowing rather than initially setting up apical polarity; they form non-polarized early cell aggregates after plating, making them unsuitable to study the early steps of polarity establishment during epithelial morphogenesis [33,37]. We therefore used MDCK (Madin-Darby canine kidney) cells, which are widely used to model epithelial polarization in several tissues [16,38]. In 3-D culture, individual MDCK cells proliferate and assemble into cyst structures, to form a polarized spherical monolayer surrounding a central lumen [39]. After 24 h of plating, MDCK cells are an ideal system to directly visualize the process of polarity establishment. At this two-cell stage, cells undergo polarity inversion, which leads to the separation of the apical cortex from the lateral cortex [16]. Consistent with our in vivo observation (Fig 3D), HTT was localized predominantly at the apical cell cortex and tight junctions (Fig 4A). It was also present in the cytoplasm, where it was enriched in cytoplasmic vesicular-like structures that colocalized with PAR3 and aPKC (Figs 4A, arrowheads, 4B, S5A and S5B, asterisks).

We examined the extent to which HTT influences the apical translocation of PAR3 and aPKC during the first steps of lumen formation. We used lentiviral short hairpin RNAs (shRNA) to stably knock down HTT expression in MDCK cells. HTT expression was efficiently impaired with two lentiviruses (shHTT1 and shHTT2) expressing shRNAs targeting different sequences of canine HTT (Fig 4C). As expected [16], we found that PAR3 and aPKC in control cysts were enriched at the apical surface, whereas the adherens junction marker β-catenin was restricted to the lateral cortex (Fig 4D). The depletion of HTT impaired the cortical accumulation of PAR3-aPKC, which displayed diffuse cytoplasmic localization (Fig 4D–4F). This impaired the transition from unpolarized epithelial cell aggregates to the establishment of the luminal PAP, where apical and basolateral plasma membranes are separated. Moreover, HTT-depleted cells displayed aberrant β-catenin localization, indicating altered specification of the basolateral cortex. Next, we introduced a construct encoding a full-length HTT tagged with mCherry (HTTFL; Fig 4C) [40]. The shHTT2 construct was designed to inhibit the expression of endogenous HTT but had no effect on the expression of exogenous HTTFL (Fig 4C) [40]. The expression of HTTFL restored the apical translocation of PAR3-aPKC and the lateral localization of β-catenin was similar to that observed in cells expressing endogenous HTT (Fig 4D–4F). Thus, HTT is instrumental for apical localization of PAR3-aPKC during the first step of polarity establishment.

We sought to investigate how HTT-mediated apical localization of PAR3-aPKC affects cystogenesis in MDCK cells. On day 4 of 3-D culture, most control cysts contained well-polarized cells that were organized around a central, single lumen (75% ± 2.74% of cysts; Fig 4G–4I). PAR3 and aPKC were localized at the apical cortex and at tight junctions, and E-cadherin was restricted to the lateral compartment (Fig 4G). Only 31.5% ± 1.3% of cells expressing shHTT1 and 28.12% ± 3.41% of cells expressing shHTT2 formed normal structures, and most cysts contained several lumens and were significantly bigger than control cysts (Fig 4G–4J). In HTT-depleted cysts, PAR3 and aPKC showed altered apical localization and abnormal colocalization with E-cadherin (Fig 4G). Remarkably, the ectopic expression of HTT in HTT-depleted cysts restored normal cystogenesis and led to the apical accumulation of PAR3 and aPKC and the lateral localization of E-cadherin (Fig 4G). By contrast, the expression of green fluorescent protein (GFP)-tagged PAR3 in shHTT2-expressing cells was not sufficient to rescue cystogenesis (Fig 4H–4J). In this context, both PAR3-GFP and aPKC showed diffuse staining in the cytoplasm (colocalization in white; Fig 4H). These observations suggest that HTT may act upstream from PAR3 to ensure the apical accumulation of PAR3-aPKC and proper cystogenesis.

### Huntingtin Coordinates Microtubule-Dependent Apical Vesicle Trafficking in 3-D Culture

**Fig 5:**
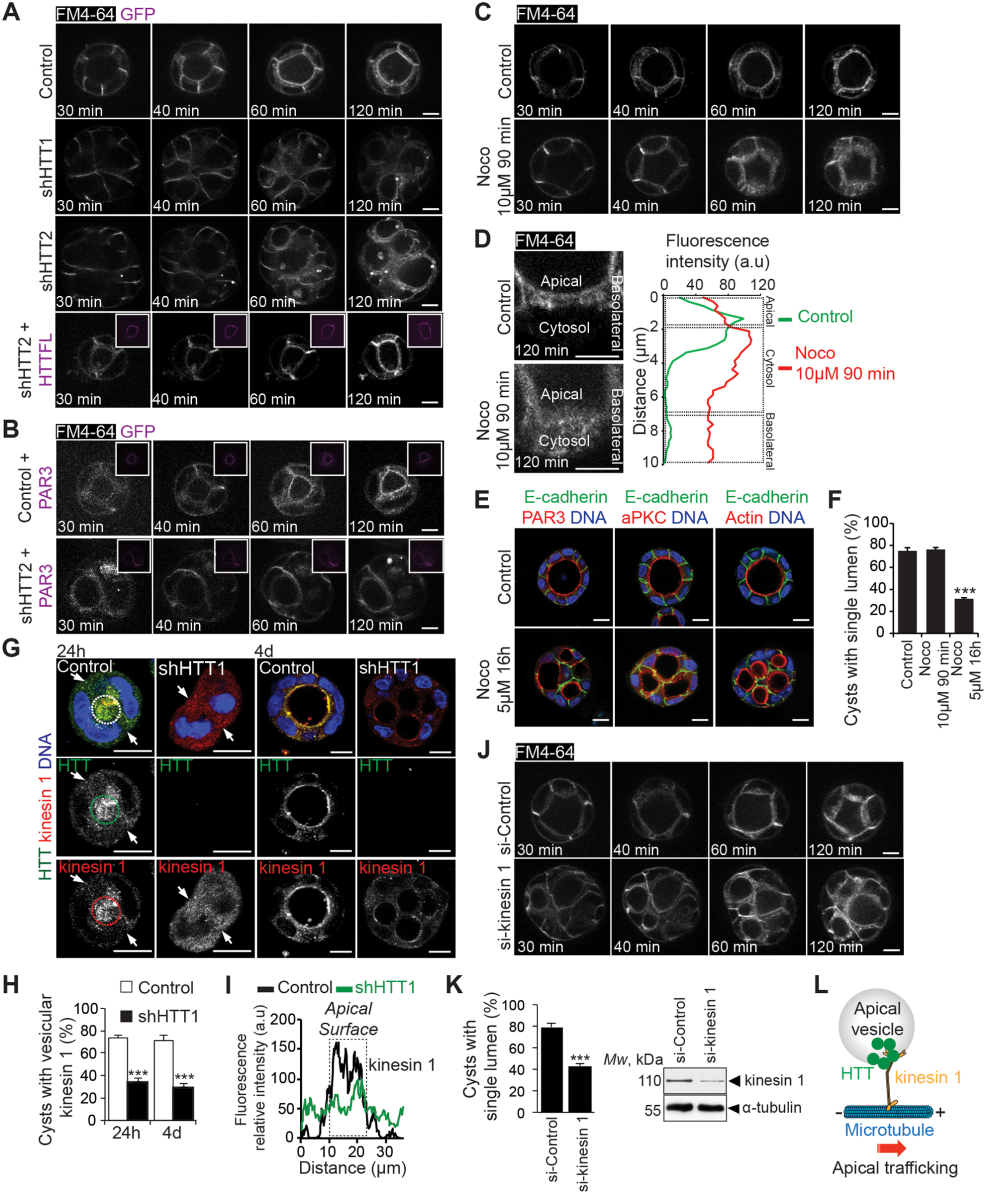
HTT regulates apical vesicular trafficking in a microtubule-dependent manner. (A–C) FM64-4 4-day MDCK 3-D structures were video-recorded. Maximum intensity and z projections are shown. Magnifications are shown in (D) (left; 120 min). (D) Representative line-scan analysis (relative fluorescence intensity; at least 20 cells were analyzed per condition). (E) Four-day MDCK 3-D structures stained for E-cadherin and PAR3, aPKC, or F-actin. (F) Percentage of acini with normal lumen (control: *n* = 94 acini, Noco 10 μM 90 min: *n* = 67 acini, Noco 5 μM 16h: *n* = 72 acini). (G) Twenty-four–hour and four-day MDCK 3-D structures stained for HTT and kinesin 1. Arrows indicate the basolateral compartment, and dashed ellipses indicate the apical surface. Colocalization of HTT and kinesin 1 is displayed in yellow (merge). (H) Percentage of 3-D structures with vesicular kinesin 1 staining (control: *n* = 22 24h-acini and *n* = 26 day 4-acini, shHTT1: *n* = 25 24h-acini and *n* = 25 day 4-acini). (I) Representative line-scan analysis (relative fluorescence intensity; at least 20 cells were analyzed per condition). (J) FM64-4 4-day MDCK 3-D structures were video-recorded. Maximum intensity and z projections are shown. (K) Left: percentage of acini with normal lumen (si-Control: *n* = 30 acini, si-kinesin 1: *n* = 28 acini), right: western blotting of MDCK cell extracts. (L) Illustration showing HTT and kinesin 1 during microtubule-dependent apical vesicular trafficking. All scale bars, 10 μm. Error bars, SEM. *** *p*<0.001. Complete statistical analyses with number of measures are detailed in S1 Data.

Apical vesicle trafficking during lumen morphogenesis depends on microtubule transport driven by motor proteins (reviewed in [1–3]). HTT interacts with microtubule-based motors to promote vesicular transport in neurons [20,23,24,41]. We thus analyzed the role of HTT in the dynamics of apical vesicles (Fig 5). We performed live-cell imaging in 3-D culture with the lipophilic dye FM4-64 [39]. In control cysts, the basolateral membrane was labeled 30 min after the addition of the dye (Fig 5A; S1 Movie). Two hours post–dye addition, both the apical membrane and the intracellular endocytic vesicles (which accumulate underneath the apical surface) were labeled (Fig 5A; S1 Movie; see also magnification in Fig 5D). By contrast, in HTT-depleted cysts treated with FM4-64, 2 h post-dye addition, endocytic vesicles failed to reach the apical membrane, accumulated in the cytoplasm, and the apical membrane was not labeled (Fig 5A; S2 and S3 Movies). The ectopic expression of HTT in shHTT1/2-expressing cysts restored normal apical vesicular trafficking and cystogenesis (Fig 5A; S4 Movie). However, the ectopic expression of PAR3 failed to do so (Fig 5B; S5 and S6 Movies), reinforcing the hypothesis that HTT is upstream from the apical vesicular trafficking machinery. The trafficking defect observed in absence of HTT correlated with aberrant cystogenesis, suggesting that HTT could mediate its effect on cystogenesis, at least in part, by regulating apical vesicular trafficking.

We then confirmed that cystogenesis was dependent on the integrity of the microtubule network and on molecular motors. We treated cysts with 10 μM of nocodazole for 90 min prior to the analysis of the trafficking of FM4-64-containing apical vesicles. Nocodazole treatment altered apical vesicle dynamics, and the vesicles accumulated in the cytoplasm (Fig 5C and 5D; S7 and S8 Movies). Moreover, treatment with 5 μM nocodazole for 16 h impaired the apical accumulation of PAR3-aPKC and led to defects in cystogenesis (Fig 5E and 5F). HTT interacts with the microtubule motor kinesin 1 to promote anterograde vesicular trafficking in neurons [24]. HTT also interacts with kinesin 1 to deliver the dynein/dynactin/NUMA/LGN complex along astral microtubules to the cell cortex during mitotic spindle orientation in mammary cells [30]. We asked whether HTT and kinesin 1 could act together during apical trafficking. Consistent with this idea, HTT and kinesin 1 colocalized and showed a punctate staining in control 24 h and day 4 3-D cultures (Figs 5G–5I and S5C; asterisks). HTT depletion disrupted kinesin 1 localization (Fig 5G–5I). Furthermore, kinesin 1 participated in apical trafficking: kinesin 1 depletion impaired the trafficking of FM4-64-containing apical vesicles, which correlated with defective cystogenesis (Fig 5J and 5K; S9 and S10 Movies). Interestingly, the trafficking defects observed in the presence of nocodazole and in absence of HTT or kinesin 1 were associated to similar aberrant cystogenesis.

Overall, these results show that HTT regulates apical vesicular trafficking (Fig 5L). Our data also support the hypothesis that this may occur through a microtubule-based, kinesin 1-dependent process.

### Huntingtin Coordinates Apical Vesicular Trafficking through RAB11A

**Fig 6:**
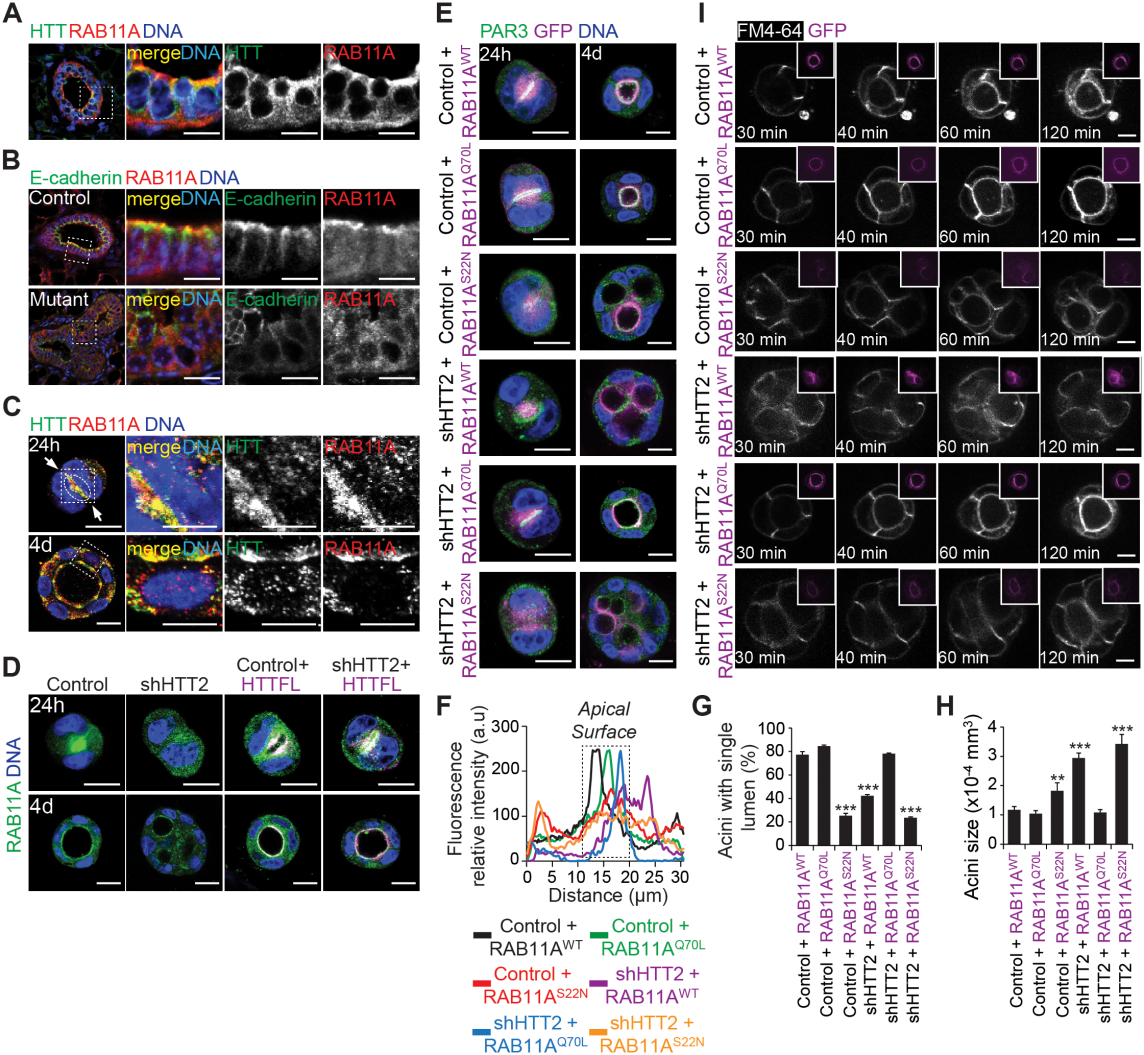
HTT colocalizes with RAB11A and regulates RAB11A activity during apical vesicular trafficking. (A) Mammary gland section stained for HTT 4C8 and RAB11A. (B) Mammary gland section stained for E-cadherin and RAB11A. (C) Twenty-four–hour and four-day MDCK 3-D cultures stained for HTT 4C8 and RAB11A. Colocalization of HTT and RAB11A is displayed in yellow (merge). (D) Twenty-four–hour and four-day MDCK 3-D cultures stained for RAB11A. HTTFL is tagged with mCherry and fluorescence is displayed in magenta and the colocalization of RAB11A and HTTFL appears in white. (E) Twenty-four–hour and four-day MDCK 3-D cultures transfected with RAB11A^WT^, RAB11A^Q70L^ or RAB11A^S22N^, stained for PAR3. RAB11 is tagged with GFP and fluorescence is displayed in magenta. The colocalization of aPKC and RAB11A appears in white. (F) Representative line-scan analysis (relative fluorescence intensity; at least 20 cells were analyzed per condition). (G) Percentage of acini with normal lumen. (H) Quantification of acini size. (G and H) Control+RAB11A^WT^: *n* = 59 acini, Control+RAB11A^Q70L^: *n* = 54 acini, Control+RAB11A^S22N^: *n* = 66 acini, shHTT2+RAB11A^WT^: *n* = 60 acini, shHTT2+RAB11A^Q70L^: *n* = 72 acini, shHTT2+RAB11A^S22N^: *n* = 93 acini. (I) FM64-4 4-day MDCK 3-D structures were video recorded. Maximum intensity and z projections are shown. All scale bars, 10 μm. Error bars, SEM. ** *p*<0.01; *** *p*<0.001. Complete statistical analyses with number of measures are detailed in S1 Data.

HTT binds RAB11A and regulates its activity in neurons [25]. RAB11 participates in lumen formation in mammalian 3-D cultures [16,42]. We hypothesized that HTT regulates apical vesicle trafficking through a RAB11A-dependent mechanism. RAB11A coimmunoprecipitated with HTT-PAR3-PAR6-aPKC (Fig 3E) and HTT localized with RAB11A at the apical membrane of mammary epithelial cells in vivo (Fig 6A). Similarly, in 24 h and 4 d 3-D cultures, HTT localized with RAB11A, showing a punctate staining which was consistent with localization on vesicles and accumulated at the apical membrane (Figs 6C and S5D; asterisks). The apical accumulation of RAB11A was impaired when HTT was depleted in LCs in vivo (Fig 6B) or in 3-D cultures of MDCK cells (Fig 6D). In shHTT2-expressing cysts, HTTFL was sufficient to restore the apical accumulation of RAB11A (Fig 6D). Thus, HTT and RAB11A both localize at the apical membrane, and HTT is required for the apical localization of RAB11A.

We then expressed different variants of GFP-tagged RAB11A in MDCK cells and analyzed the apical targeting of PAR3 and the subsequent effects on cystogenesis. In control cysts at 24 h, wild-type RAB11A (RAB11A^WT^) and the constitutively active RAB11A^Q70L^ were localized, along with endogenous PAR3, at the apical surface (Fig 6E and 6F). By contrast, the dominant-negative RAB11A^S22N^ accumulated in the cytoplasm and impaired the apical accumulation of PAR3. Cystogenesis was altered at day 4 in cysts expressing RAB11A^S22N^, whereas the expression of RAB11A^WT^ or RAB11A^Q70L^ mostly resulted in cysts with a single lumen (Fig 6E and 6G). We next analyzed whether the expression of the RAB11A variants rescues the defects in the apical targeting of PAR3 and cystogenesis induced by the loss of HTT. Remarkably, in contrast with RAB11A^WT^ and RAB11A^S22N^, RAB11A^Q70L^ expression in shHTT2-treated cysts was sufficient to rescue the apical translocation of PAR3 and cystogenesis (Fig 6E and 6H). We obtained similar results with aPKC (S6 Fig). We conclude that HTT regulates RAB11A to coordinate the apical vesicular trafficking of PAR3-aPKC.

We then analyzed apical trafficking by live cell imaging of FM4-64-containing vesicles. The accumulation of FM4-64-containing vesicles at the apical surface was higher in control cysts expressing RAB11A^Q70L^ than in those expressing exogenous RAB11A^WT^ (Fig 6I; S11 and S12 Movies). RAB11A^S22N^ expression altered apical vesicle trafficking, which correlated with marked defects in cystogenesis (Fig 6I; S13 Movie). In HTT-depleted cysts, RAB11A^Q70L^ was able to recover FM4-64-apical vesicle trafficking and normal cystogenesis, whereas both RAB11A^WT^ and RAB11A^S22N^ failed to do so (Fig 6I; S14–S16 Movies). These observations show that HTT is instrumental for RAB11A-mediated apical vesicular trafficking.

## Discussion

**Fig 7:**
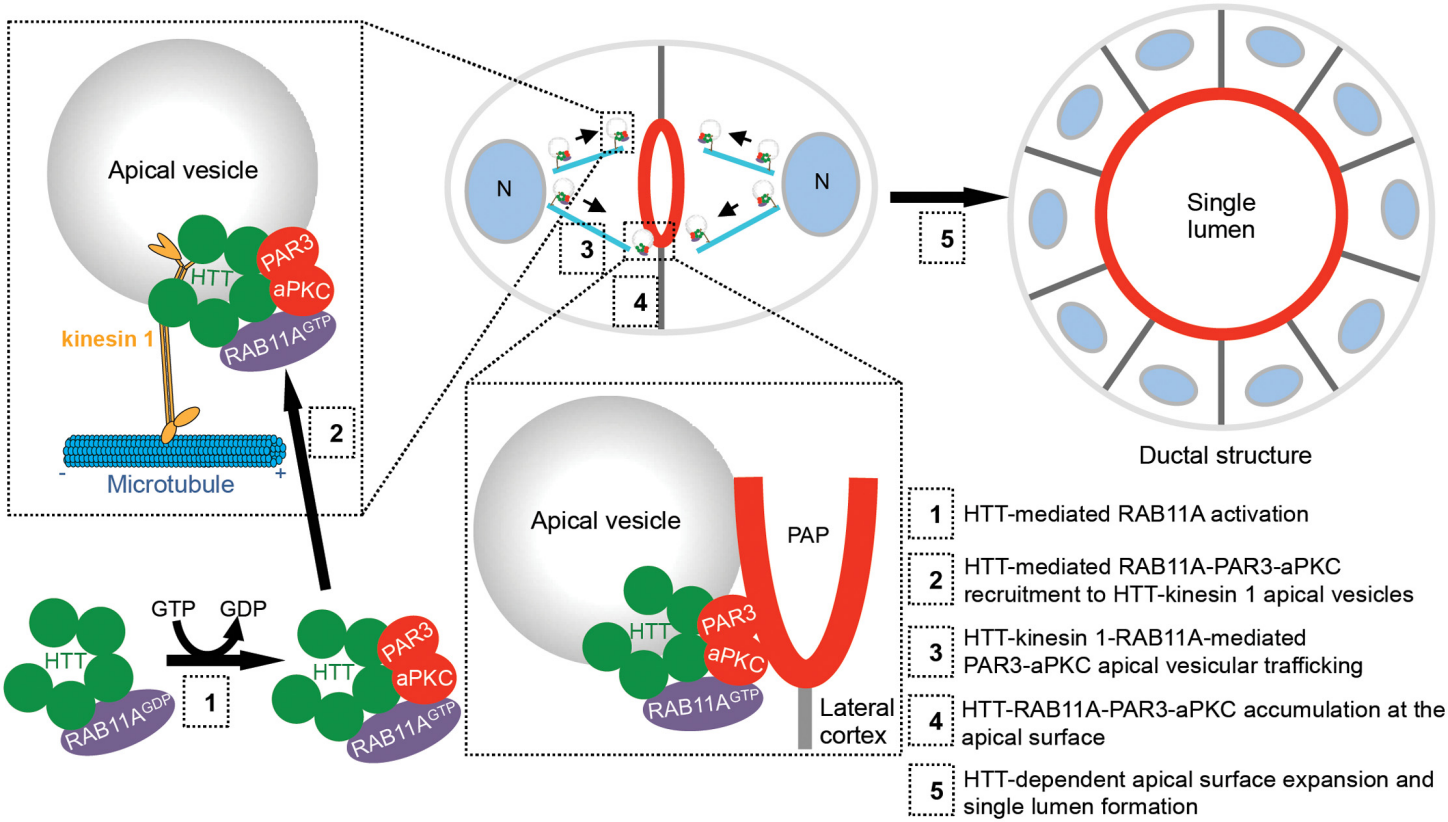
Model for HTT-mediated regulation of apical polarity. During epithelial morphogenesis, HTT modulates the activation of RAB11A (1). HTT-RAB11A forms a complex with PAR3-aPKC, which may be recruited to HTT-kinesin 1 apical vesicles (2). HTT coordinates apical recycling of PAR3-aPKC vesicles (3). PAR3-aPKC accumulation at the pre-apical patches (PAP) (4) triggers the expansion of the apical membrane, leading to the formation of a central lumen (5).

In this study, we propose a model in which HTT regulates RAB11A-mediated apical trafficking of the PAR-polarity complex in the mammary epithelium, with consequences for lumen formation and tissue architecture (Fig 7). Interestingly, loss of any of the components of the CDC42-PAR6-PAR3-aPKC complex also causes the formation of multiple lumens and thereby alters epithelial morphogenesis [10,43]. Disruption of the interaction between PAR3 and aPKC in the mammary gland induces malformations during mammary gland morphogenesis [15]. Remarkably, the epithelial architectural defects induced by the loss of HTT persisted during pregnancy and lactation and affected functional differentiation and milk production. Consistent with these findings, the expression of apical polarity proteins is essential for the differentiation of alveolar cells to milk secreting units [44].

We recently showed that the depletion of HTT from the basal compartment of the mammary gland alters luminal cell polarity [30]. In the K5*Cre*; *Htt* ^*flox/flox*^ mouse model used in this study, HTT was depleted from basal cells but also partially from LCs. Thus, we were unable to conclude whether the effect of HTT on luminal polarity was direct or indirect. Here, we specifically removed HTT from LCs because HTT is strongly expressed in these cells and LCs are highly polarized. We show that HTT is important for the establishment of apical polarity during mammary morphogenesis. We provide evidence that one of the mechanisms by which HTT mediates its effect is the regulation of the apical trafficking of PAR3-aPKC. However, we cannot exclude that loss of HTT may lead to altered cell organization by another mechanism that would subsequently lead to a polarization defect. In particular, how HTT-dependent vesicular trafficking coordinates the segregation between apical and basolateral compartments remains to be determined. Early work in *Drosophila melanogaster* identified a Rab11-dependent trafficking of E-cadherin essential for epithelial junction maturation [45]. Furthermore, HTT forms a complex with β-catenin [46]. It is then tempting to speculate that HTT may also regulate basolateral trafficking through RAB11A during polarity establishment.

The orientation of mitosis also regulates lumen formation; therefore, alteration in this process may also contribute to the phenotypes observed. Indeed, HTT regulates spindle orientation in MaSCs and controls the cortical accumulation of the mitotic complex, including LGN, NUMA, dynein, and dynactin [30]. Interestingly, RAB11A, PAR3, and aPKC are also involved in spindle orientation [47,48]. Thus HTT could help localize RAB11A, PAR3, and aPKC during lumen formation and mammary epithelium morphogenesis to ensure the coordination of spindle orientation and apical trafficking.

RAB proteins cycle between GDP bound (inactive) and GTP bound (active) states and these cycles are controlled by guanine nucleotide exchange factors (GEFs) and GTPase-activating proteins (GAPs). In their active form, RABs are associated with membranes and carry out their functions though effector partner proteins. RAB11 controls vesicle trafficking in apical recycling endosomes and is necessary for epithelial morphogenesis [17,18]. Our results suggest that HTT acts upstream from PAR3 by regulating RAB11 activity. These results are consistent with a previous study showing that HTT binds RAB11A and regulates its activity in neurons [25]. The authors of this study showed that the inhibition of HTT expression affects the attachment of RAB11 to membranes and the guanine nucleotide exchange activity on RAB11. They also showed that HTT binds RAB11-GDP preferentially, suggesting that HTT either acts as a GEF for RAB11 or activates GEF activity on RAB11. Nonetheless, other mechanisms besides the microtubule-based apical delivery of polarity proteins may be affected by the HTT-mediated regulation of RAB11 activity. Indeed, a recent study demonstrated that RAB11 localizes recycling endosomes to mitotic spindle poles by dynein-mediated transport [48]. Similarly, during mitosis, the interaction of HTT with dynein is required for the localization of spindle pole proteins [26,30].

The actin and the microtubule cytoskeletons and their associated motor proteins are critical for apical vesicle trafficking during lumen morphogenesis (reviewed in [2,3]). Interestingly, previous studies suggest that HTT is a crucial link between the microtubule and the actin cytoskeletons. HTT forms a complex with dynein, dynactin, and kinesin 1 (KIF5) in neurons to promote retrograde and anterograde microtubule-based axonal transport of several cargoes [19–24]. RAB11-containing vesicles are bidirectionally transported by HTT in vivo in whole-mount *Drosophila* larval axons [49]. During mitosis, HTT mediates the cortical localization of dynein, dynactin, LGN, and NUMA through kinesin 1-dependent transport along astral microtubules [30]. Here, we suggest that HTT acts with kinesin 1 to coordinate microtubule-based apical trafficking in a RAB11A-dependent pathway. The early endosomal trafficking effector, RAB5, binds HTT through HAP40, and RAB8, which associates with the Golgi membrane, can also form a complex with HTT through the myosin VI linker, optineurin [50,51]. The HAP40-HTT complex also interacts with optineurin [52]. Thus, HTT may regulate actin-dependent dynamics when in complex with HAP40-Optineurin-MyosinVI, and it may regulate microtubule-dependent transport when in complex with dynein-dynactin-kinesin.

Finally, the cell polarity machinery is perturbed during tumorigenesis with consequences for metastasis. For instance, PAR3 levels are significantly lower in human breast cancers than in non-malignant tissue, and this down-regulation correlates with the overactivation and mislocalization of aPKC [53,54]. In murine models of breast cancer, loss of PAR3 promotes breast tumorigenesis and metastasis [54]. Thus, the identification of new regulators of the apical vesicle trafficking machinery is critical for our understanding of both normal development of the epithelium and pathogenic pathways leading to metastasis.

## Materials and Methods

### Constructs and siRNAs

pARIS-mCherry-HTTQ23 (referred to herein as Q23HTTFL) was previously described [40]. GFP-RAB11A wild-type (WT), dominant-negative (S25N), and constitutively active (Q70L) (referred to herein as RAB11A^WT^, RAB11A^S25N^ and RAB11A^Q70L^, respectively) were obtained from B. Goud (Institut Curie, France). PAR3-GFP (referred to herein as PAR3) was provided by Dr. I. Mellman (Genentech, CA, United States) [55]. si-kinesin 1-sens (5′-GCAGUCAGGUCAAAGAAUA-3′) and si-kinesin 1-antisens (5′-UAUUCUUUGACCUGACUGC-3′) were used for siRNA against mouse/rat/human KIF5B (si-kinesin 1). siRNA negative control (si-Control) from Eurogentec (OR-0030-neg05) was used.

### Cell Lines and Transfection

MCF-10A, a spontaneously immortalized, nontransformed human mammary epithelial cell line derived from the breast tissue [56] was maintained in DMEM/F12 (Invitrogen, Carlsbad, CA) supplemented with 5% donor horse serum, 20 ng/ml EGF (Peprotech, Rocky Hill, NJ), 10 μg/ml insulin (Sigma, St Louis, MO), 1 ng/ml cholera toxin (Sigma), 100 μg/ml hydrocortisone (Sigma), 50 U/ml penicillin, and 50 μg/ml streptomycin (Invitrogen) at 37°C in a humidified 5% CO_2_ atmosphere.

MDCK cells were maintained in DMEM (Invitrogen) supplemented with 10% fetal calf serum, 50 U/ml penicillin and 50 μg/ml streptomycin (Invitrogen) at 37°C in a humidified 5% CO2 atmosphere.

Cells were spread in 10 cm^2^ plate and transfected using Lipofectamin 2000 (Invitrogen). After 24 h, cells were plated on Matrigel for 3-D cultures. Alternatively, after 48–72 h, cells were lysed or fixed and immunoprocessed.

Three-dimensional cultures of MCF-10A and MDCK cells in Matrigel were performed as described previously [33]. In brief, MCF-10A and MDCK cells were trypsinized and resuspended to single cell suspension of 2 x 10^4^ cells/ml (MCF-10A) and 4 x 10^4^ cells/ml (MDCK) in 2% Matrigel (BD). Four-hundred μl of cells were plated in each well of 8-well Lab-Tek II chamber slides (Thermo Fisher Scientific) precovered with matrigel (25 μl per well). MCF-10A cells were fed every 4 d and grown for 8–20 d. MDCK cells were fed every 2 d and grown for 1–4 d.

### Lentivirus Production and Infection

Stable knockdown of HTT in MDCK cells was done as previously described for LGN [57]. Oligos containing target sequences were cloned in the pLKO.1 vector. HEK293 cells were transfected with the RNAi vectors and the lenti-packaging mix (Invitrogen). Virus supernatant was collected 48 h after transfection and used to infect MDCK cells (plated in 12-well plates and transferred to P-100 plates 24 h after infection). Clones of interest were selected using puromycin (5 μg/ml) and isolated 1 wk later. Target sequences for dog HTT were 5′-GTGCCTCAACAGAGTCATAA-3′ (shHTT1) and 5′-GGTTACAGTTAGAACTCTATA-3′ (shHTT2). Empty pLKO.1 was used as a control.

Lentivirus-mediated stable knockdown of HTT in MCF-10A cells was described elsewhere [58]. Briefly, shRNA targeting the human HTT recognized a region within exons 8–9 and was transcribed from the polymerase III H1 promoter 5′-AGCTTTGATGGATTCTAA-3′ (sh-HTT). The sh-Control recognized a sequence within the firefly luciferase gene 5′-CGTACGCGGAATACTTCGA-3′. EGFP reporter gene under the control of the mouse PGK promoter allowed the selection of positive clones.

The knockdown efficiency was analyzed by immunoblotting and immunostaining of HTT.

### Drug Treatment

Drugs were dissolved in DMSO and kept at -20°C as 10 mM stock solutions. To depolymerize microtubules, MDCK cells were treated with 10 μM or 5 μM nocodazole (Sigma) for 90 min or 16 h respectively. For live-imaging, MDCK cells were treated with 4 μM FM4-64 Dye (*N*-(3-Triethylammoniumpropyl)-4-(6-(4-(Diethylamino) Phenyl) Hexatrienyl) Pyridinium Dibromide) (Life Technology) for 30 min.

### Antibodies and Immunostaining Procedures

Anti-HTT antibodies used in this study were previously described: mAb 4C8 (epitope 445–456, clone HU-4C8-As, Euromedex), mAb D7F7 (Cell Signaling) [26].

For immunofluorescence, the primary monoclonal antibodies used were: anti-ß-catenin (1:200; BD Bioscience), anti-GM130 (1:100; BD Bioscience) and anti-HTT 4C8. The primary polyclonal antibodies used were: anti-PAR3 (1:200; Chemicon), anti-aPKC (1:200; Santa-Cruz Biothechnology), anti-RAB11A AT15 (1:200; Abcam) and anti-cleaved caspase 3 (1:100; Cell Signaling). Rhodamin-conjugated Phalloidin was used for cortical actin (F-actin) labeling (Molecular Probes). Secondary antibodies used were goat anti-mouse and anti-rabbit conjugated to AlexaFluor-488 or AlexaFluor-555 (Molecular Probes) at 1:200.

MCF-10A cells grown on chamber slides were fixed in 2% paraformaldehyde at room temperature for 20 min. Cells were washed three times in PBS:glycine (130 mM NaCl, 7 mM Na_2_HPO_4_, 3.5 mM NaH_2_PO_4_, 100 mM glycine; 15 min each), blocked first in IF buffer (130mM NaCl, 7mM Na_2_HPO_4_, 3.5mM NaH_2_PO_4_, 7.7 mM NaN_3_, 0.1% BSA, 0.2% Triton X-100, 0.05% Tween 20) containing 10% goat serum (1–2 h) and then with a second blocking buffer (IF buffer containing 10% goat serum and 20 μg/ml goat anti-mouse F(ab′)2; Jackson Immunoresearch) for 30–45 min. Anti-cleaved caspase 3, anti-β-catenin or anti-GM130 were diluted in the second blocking buffer and incubated overnight at 4°C. Acini were stained with anti-rabbit AlexaFluor-555.

MDCK cells grown on chamber slides were fixed with 4% paraformaldehyde in PBS and permeabilized with 0.5% Triton X-100 in PBS. Fixed cells were blocked with 10% normal goat serum/1%BSA in PBS for 2 h, and then incubated with anti-ß-catenin and anti-PAR3 or anti-aPKC overnight at 4°C. Alternatively cells were incubated with anti-HTT 4C8 and anti-PAR3, anti-aPKC or anti-RAB11A 3 h at RT. Cells were stained with anti-mouse and anti-rabbit AlexaFluor-488 or AlexaFluor-555. Cysts with actin staining at the apical surface of cells surrounding a single lumen were identified as cysts with normal lumens.

For all immunostainings, the slides were counterstained with DAPI (Roche) and mounted in Mowiol. The pictures were captured with a Leica SP5 laser scanning confocal microscope equipped with a X63 oil-immersion objective. *Z*-stack steps were of 0.5 μm. Images were treated with ImageJ (http://rsb.info.nih.gov/ij/, NIH, US).

### Quantification and Image Analyses

To measure the relative fluorescence intensity at the apical surface, a 30-pixel line was drawn across the apical surface and the cytoplasm using ImageJ software. The Line Scan function of ImageJ was used to reveal the relative fluorescence intensity across the line. The quantification of the polarization of the Golgi in MCF-10A 3-D acini was done using a home-built macro (ImageJ software, see below for details).

### Live-Cell Microscopy

For live-cell imaging, MDCK cells were grown for 4 d in 24 mm Matrigel-coated coverglass, mounted in 6-well plate (TPP). 30 min before observation, acini were incubated in culture media containing 4 μM FM4-64. Imaging was performed at 37°C in 5% CO_2_ using an inverted microscope (Eclipse T*i*; Nikon) with a 60 x 1.42 NA oil immersion objective coupled to a spinning-disk confocal system (CSU-X1; Yookogawa) fitted with an EM-CCD camera (Evolve; Photometrics). Exposure times were 200 msec and 10% laser power. Image stacks of 50 planes spaced 1 μm apart were taken at six stage positions every 5 min for 2 h. Maximum intensity projection of the fluorescent channels was performed. Images were treated with ImageJ.

### Cell Extracts, Immunoblotting, and Immunoprecipitation Experiments

MCF-10A and MDCK cells were lysed in NP40 buffer (50 mM Tris, pH 7.4, 250 mM NaCl, 5 mM EDTA, 50 mM NaF, 1 mM Na_3_VO_4_, 1% Nonidet P40 (NP40), 0.02% NaN_3_) and centrifuged at 11,000 x g for 10 min at 4°C. MCF-10A 3-D cultures were treated with trypsin 0.25% for 15 min to break the Matrigel, then acinar structures were washed with PBS1X and resuspended in NP40 lysis buffer, containing protease inhibitor cocktail (Sigma), and centrifuged at 11,000 x g for 10 min at 4°C. 20–30 μg of protein extracts were loaded onto SDS-PAGE (polyacrylamide gel electrophoresis) and subjected to Western blot analysis. Primary monoclonal antibodies used were: anti-HTT 4C8 (1:3,000), anti-HTT D7F7 (1:500), anti-α-tubulin (1:5,000). Primary polyclonal antibodies used were: anti-PAR3 (1:1,000), anti-PAR6 (1:500), anti-aPKC (1:1,000), anti-RAB11A (1:500) and anti-mCherry (1:1,000; Institut Curie, Paris). Secondary antibodies used were HRP-conjugated goat anti-mouse/anti-rabbit (1:10,000; Amersham).

For immunoprecipitations, MCF-10A cells were lysed in IP buffer (Tris 50 mM pH 7.4, 250 mM NaCl, 5 mM EDTA, 50 mM NaF, 1% Na_3_VO_4_, 1% NP40, 0.02% NaN_3_, 50mM KH_2_PO_4_) containing protease inhibitor cocktail. Lysates (500 μg at 1 μg/μl) were precleared 1 h at 4°C with 50 μl of a 50% solution of protein A or G beads. Extracts were incubated for 1 h at 4°C with 5 μg of anti-HTT (4C8) antibody or anti-PAR3 prebound with 50 μl of a 50% solution of protein A or G sepharose beads (Sigma). Beads were washed three times with IP buffer. Bound proteins were eluted with SDS loading buffer, resolved by SDS-PAGE and subjected to immunoblotting analysis.

### Mouse Strains

Mice expressing the Cre recombinase under the control of the MMTV promoter (MMTV*Cre*) and *Htt* ^*flox/flox*^ mice were previously described [31,32]. All mice were bred in a C57BL6 genetic background. *Htt* ^*flox/flox*^ mice were used as controls and MMTV*Cre*;*Htt* ^*flox/flox*^ as mutants. All experiments were performed in strict accordance with the recommendations of the European Community (86/609/EEC) and the French National Committee (87/848) for care and use of laboratory animals (permissions 91–448 to SH and 76–102 to SE).

### Whole Mounts and Quantification of Ductal Morphogenesis

Whole mounts were prepared as described elsewhere [59]. Glands were fixed with MethaCarn (60% methanol, 30% chloroform, 10% glacial acetic acid; overnight, room temperature) and hydrated by incubation in ethanol solutions (100%, 70%, 50%, 30%; 15 min each) and distilled water (2 x 5 min). Mounts were then stained overnight with carmine (2%) and aluminum potassium sulphate (5%)(Sigma, Buchs, Switzerland), dehydrated in ethanol solutions (70%, 90%, 95%, and 2 x 100%; 15 min each), and cleared with xylene (overnight). Images were captured with an Epson Perfection 3200 scanner.

Mammary gland development was analyzed as described elsewhere [60]. Briefly, the degree of ductal invasion was determined by dividing the duct length by the mammary gland length from mid-point of lymph node, and the numbers of total branches and TEBs were determined on whole-mount images by the ImageJ program.

### Histology and Immunostaining

Dissected mammary fat pads were fixed in MethaCarn and embedded in paraffin. Seven μm-thick sections were deparaffinized before staining with primary antibodies (overnight, 4°C), and secondary antibodies (1 h, room temperature). Nuclei were counterstained with DAPI. Primary antibodies used were: rabbit polyclonal anti-PAR3 (1:200; Chemicon), anti-aPKC (1:200; clone C-20, Santa Cruz Biotechnology), anti-RAB11A (1:200; Abcam), anti-pSTAT5 (Tyr694, 1:100; Cell Signalling), anti-cleaved caspase 3 (1:100; Cell Signalling), anti-WAP (1:300; clone R-131, Santa Cruz Biotechnology) and anti-keratin 5 (K5) (1:2,000; Covance); rabbit monoclonal anti-KI67 (1:100; clone SP6, Neo Markers); and mouse monoclonal anti-HTT (1:300; 4C8), anti-E-cadherin (1:200; BD Bioscience) and anti-GM130 (1:100; BD Bioscience). Antigen retrieval was performed by boiling the slides for 10 min in a microwave in 10 mM citrate buffer (pH 6) for cleaved caspase 3, Ki67, WAP, and p-STAT5A, or in EDTA buffer (pH 8.8) for 10 min for PAR3, aPKC, RAB11A, HTT, GM130, K5, and E-cadherin antibodies. Secondary antibodies used were goat anti-mouse and anti-rabbit conjugated to AlexaFluor-488 or AlexaFluor-555 or Biotin (Vector Laboratories).

### Isolation of the Mammary Epithelial Cells and Flow Cytometry

The isolation of mammary epithelial cells and the separation of basal and luminal cells were done as described elsewhere [61,62]. Once mechanically dissociated, mammary fat pads were digested (90 min, 37°C) in CO2-independent medium (Invitrogen) containing 5% fetal bovine serum, 3 mg/ml collagenase (Roche Diagnostics) and 100 U/ml hyaluronidase (Sigma). Cells were resuspended in 0.25% trypsin-EDTA (1 min), and then in 5 mg/ml dispase (Roche Diagnostics) with 0.1 mg/ml DNase I (Sigma) (5 min). Red blood cells were lysed in NH_4_Cl. Basal and luminal cells were isolated from mammary epithelial cells obtained from the inguinal glands of five 12-wk-old virgin MMTV *Cre* mice. Cells were stained with the following antibodies: anti-CD24-FITC (clone M1/69; BD Pharmingen), anti-CD49F-PE (clone GoH3; BD Pharmingen), anti-CD45-APC (clone 30-F11; Biolegend) and anti-CD31-APC (clone MEC13.3; Biolegend). Basal (CD24-low/α6-high) and luminal (CD24-high/α6-low) cells were purified using FACSAria III (SORP) (Becton Dickinson).

### Quantitative RT-PCR

RNA samples were retrotranscribed using the First-Strand cDNA Synthesis Kit (Invitrogen). cDNAs were diluted 1:10 and submitted to RT-PCR with 7900HT Fast real time PCR system (Applied biosystems) using power SYBR Green PCR Master mix (Applied biosystems) with the following oligonucleotide pairs: *Htt* (5′-CTCAGAAGTGCAGGCCTTACCT-3′, 5′-GATTCCTCCGGTCTTTTGCTT-3′ and 5′-CTCAGAAGTGCAGGCCTTACCT-3′, 5′-GATTCCTCCGGTCTTTTGCTT-3′) [63], *Cre* (5′-TTCCCGCAGAACCTGAAGAT-3′, 5′- GCCGCATAACCAGTGAAACA-3′) [62], *Krt18* (5′-CGAGGCACTCAAGGAAGAAC-3′, 5′-AATCTGGGCTTCCAGACCTT-3′), *Elf5* (5′-CCAACGCATCCTTCTGTGAC-3′, 5′-AGGCAGGGTAGTAGTCTTCA-3′), *Wap* (5′-AACATTGGTGTTCCGAAAGC-3′, 5′-GGTCGCTGGAGCATTCTATC-3′), *Csn2* (5′-TGCAGGCAGAGGATGTGCTCCAGGCT-3′, 5′-GGCCTGGGGCTGTGACTGGATGCT-3′) (Primer3v.0.4.0; http://bioinfo.ut.ee/primer3-0.4.0/primer3). *β-actin* (5′-AGGTGACAGCATTGCTTCTG-3′, 5′-GCTGCCTCAACACCTCAAC-3′) and *hprt* (5′-GCTGGTGAAAAGGACCTCT-3′, 5′-CACAGGACTAGAACACCTGC-3′) [29] genes were used as internal controls. Fold changes were calculated using the ddCT method.

### Macros “Golgi Orientation in 3-D Culture”

This macro was developed on site by F.P. Cordelières at the Institut Curie Imaging Facility.

// Macro angle measurement

run("Set Measurements...", "area mean min centroid center integrated redirect = None decimal = 1");

run("Clear Results");

roiManager("reset");

run("Select None");

setTool ("freehand");

Xsel = newArray(3);

Ysel = newArray(3);

waitForUser("Draw ROI around the cyst");

run("Duplicate...", "title = duplicate duplicate");

run("Properties...", "channels = 3 slices = 1 frames = 1 unit = pixel pixel_width = 1 pixel_height = 1 voxel_depth = 1 frame = [0 sec] origin = 0,0");

run("Select None");

setTool("point");

waitForUser("Indicate the point A");

run("Measure");

Xsel[0] = getResult("X", 0);

Ysel[0] = getResult("Y",0);

run("Clear Results");

waitForUser("Indicate the point B");

run("Measure");

Xsel[1] = getResult("X", 0);

Ysel[1] = getResult("Y",0);

run("Clear Results");

Xsel[1] = (Xsel[0] +Xsel[1])/2;

Ysel[1] = (Ysel[0]+Ysel[1])/2;

run("Select None");

setSlice(3);

setAutoThreshold("Percentile dark");

run("Analyze Particles...", "size = 30000-Infinity pixel circularity = 0–1.00 show = Nothing display clear add slice");

resetThreshold();

verif = roiManager("Count");

if(verif >1) exit ("Many cysts detected");

if(verif = = 0) exit ("No detected cyst");

Xsel[2] = getResult("X", 0);

Ysel[2] = getResult("Y", 0);

roiManager("reset");

run("Clear Results");

setTool("angle");

makeSelection("angle", Xsel, Ysel);

run("Measure");

angle = getResult("Angle", 0);

if (angle >90) angle = 180—angle;

print("Measured angle is " + angle + " degree");

### Statistical analyses

GraphPad Prism 6.0 software (San Diego, CA) was used for statistical analysis. Complete statistical analyses with number of measures are detailed in S1 Data.

## Supporting Information

S1 Data Complete statistical data for Figs 1–6 and S1–S3. (DOC)

S1 Fig Loss of HTT and mammary ductal morphogenesis at 12 wk. (A) Carmine-stained whole mounts of 12-wk-old virgin mammary glands. (B) Number of branches in 12-wk-old virgin mammary glands. (C) Quantitative real-time RT-PCR analysis of *Ki67* gene in mammary epithelial cells from 12-wk-old virgin mice. Data are presented as means obtained in three independent experiments (control: three mice per experiment, mutant: three mice per experiment). Error bars, SEM.
(TIF)

S2 Fig Loss of HTT alters mammary epithelial morphogenesis in MCF10A 3-D culture. (A) Western blotting of extracts from day 6, 12, and 20 shControl and shHTT MCF-10A cells in 3-D culture. (B) Day 8, 10, and 20 shControl and shHTT MCF-10A 3-D acini stained for cleaved caspase 3. Scale bar, 10 μm. (C) Percentage of cleaved caspase 3-positive luminal cells: Day 8 (shControl: *n* = 47 acini, shHTT: *n* = 32 acini); Day 10 (shControl: *n* = 45 acini, shHTT: *n* = 30 acini); Day 20 (shControl: *n* = 31 acini, shHTT: *n* = 30 acini). (D) Day 20 shControl and shHTT MCF-10A acini stained for ß-catenin. Scale bar, 10 μm. (E) Quantification of the number of intraluminal cells in day 10 and 20 shControl and shHTT MCF-10A acini: Day 10 (shControl: *n* = 34 acini, shHTT: *n* = 35 acini); Day 20 (shControl: *n* = 38 acini, shHTT: *n* = 46 acini). (F) Quantification of day 10 and 20 shControl and shHTT MCF-10A acini size: Day 10 (shControl: *n* = 35 acini, shHTT: *n* = 35 acini); Day 20 (shControl: *n* = 38 acini, shHTT: *n* = 30 acini). Error bars, SEM. ** *p*<0.01; *** *p*<0.001.
(TIF)

S3 Fig Loss of HTT in luminal cells alters mammary epithelial morphogenesis during lactation. (A) Mammary gland sections stained for E-cadherin and PAR3 or aPKC. (B) Mammary gland sections stained for keratin 5 (K5) and GM130. (C) Percentage of LCs showing ribbon-like and fragmented GM130 (control: *n* = 3 mice; mutant: *n* = 3 mice). (D) Day 20 shControl and shHTT MCF-10A 3-D acini stained for GM130. (E) Deviation of the Golgi from acini center (α°) (shControl: *n* = 56 acini, shHTT: *n* = 67 acini). (F) Percentage of acini with Golgi uncoupled to center (shControl: *n* = 56 acini, shHTT: *n* = 67 acini). All scale bars, 10 μm; Error bars, SEM; ***p<0.001.
(TIF)

S4 Fig Loss of HTT does not affect microtubule integrity. MCF10-10A cells stained for α-tubulin. Scale bar, 10 μm.
(TIF)

S5 Fig Colocalization of HTT and PAR3, aPKC, RAB11A, and Kinesin 1. Representative line-scan analysis of overlap and non-overlap of HTT with PAR3 (A), aPKC (B), kinesin 1 (C) and RAB11A (D) (relative fluorescence intensity; at least 20 cells were analyzed per condition). Asterisks indicate colocalizations.
(TIF)

S6 Fig HTT regulates RAB11A for aPKC apical vesicular trafficking. (A) Twenty-four–hour MDCK 3-D cultures transfected with RAB11AQ70L, RAB11AWT, or RAB11AS22N, stained for aPKC. RAB11A is tagged with GFP, and fluorescence is displayed in magenta, and the colocalization of aPKC and RAB11A appears in white. (B) Representative line-scan analysis (relative fluorescence intensity; at least 20 cells were analyzed per condition).
(TIF)

S1 Movie FM4-64 apical trafficking in control cells. Control MDCK acini were treated with FM4-64 for 30 min. Images corresponding to 50 planes spaced by 0.6 μm through the cell volume were collected every 5 min using a spinning-disk confocal microscope (CSU-X1; Yookogawa). Maximum intensity projections are shown over time.
(AVI)

S2 Movie Loss of HTT alters FM4-64 apical trafficking. shHTT1 MDCK acini were treated with FM4-64 for 30 min. Images corresponding to 50 planes spaced by 0.6 μm through the cell volume were collected every 5 min using a spinning-disk confocal microscope (CSU-X1; Yookogawa). Maximum intensity projections are shown over time.
(AVI)

S3 Movie Loss of HTT alters FM4-64 apical trafficking. shHTT2 MDCK acini were treated with FM4-64 for 30 min. Images corresponding to 50 planes spaced by 0.6 μm through the cell volume were collected every 5 min using a spinning-disk confocal microscope (CSU-X1; Yookogawa). Maximum intensity projections are shown over time.
(AVI)

S4 Movie Ectopic expression of HTT restores loss of HTT-induced defect in FM4-64 apical trafficking. shHTT2 MDCK acini were transfected with HTTFL and treated with FM4-64 for 30 min. Images corresponding to 50 planes spaced by 0.6 μm through the cell volume were collected every 5 min using a spinning-disk confocal microscope (CSU-X1; Yookogawa). Maximum intensity projections are shown over time.
(AVI)

S5 Movie FM4-64 and PAR3 apical trafficking in control cells. Control MDCK acini were transfected with PAR3-GFP and treated with FM4-64 for 30 min. Images corresponding to 50 planes spaced by 0.6 μm through the cell volume were collected every 5 min using a spinning-disk confocal microscope (CSU-X1; Yookogawa). Maximum intensity projections are shown over time.
(AVI)

S6 Movie PAR3 is not sufficient to restore loss of HTT-induced defect in FM4-64 apical trafficking. shHTT2 MDCK acini were transfected with PAR3-GFP and treated with FM4-64 for 30 min. Images corresponding to 50 planes spaced by 0.6 μm through the cell volume were collected every 5 min using a spinning-disk confocal microscope (CSU-X1; Yookogawa). Maximum intensity projections are shown over time.
(AVI)

S7 Movie FM4-64 apical trafficking in control cells. Control MDCK acini were treated with FM4-64 for 30 min and DMSO for 90 min. Images corresponding to 50 planes spaced by 0.6 μm through the cell volume were collected every 5 min using a spinning-disk confocal microscope (CSU-X1; Yookogawa). Maximum intensity projections are shown over time.
(AVI)

S8 Movie Microtubule disassembly alters FM4-64 apical trafficking. Control MDCK acini were treated with FM4-64 for 30 min and 10 μM nocodazole for 90 min. Images corresponding to 50 planes spaced by 0.6 μm through the cell volume were collected every 5 min using a spinning-disk confocal microscope (CSU-X1; Yookogawa). Maximum intensity projections are shown over time.
(AVI)

S9 Movie FM4-64 apical trafficking in control cells. si-Control MDCK acini were treated with FM4-64 for 30 min. Images corresponding to 50 planes spaced by 0.6 μm through the cell volume were collected every 5 min using a spinning-disk confocal microscope (CSU-X1; Yookogawa). Maximum intensity projections are shown over time.
(AVI)

S10 Movie Loss of kinesin 1 alters FM4-64 apical trafficking. si-kinesin 1 MDCK acini were treated with FM4-64 for 30 min. Images corresponding to 50 planes spaced by 0.6 μm through the cell volume were collected every 5 min using a spinning-disk confocal microscope (CSU-X1; Yookogawa). Maximum intensity projections are shown over time.
(AVI)

S11 Movie FM4-64 and RAB11A^WT^ apical trafficking in control cells. Control MDCK acini were transfected with RAB11A^WT^-GFP and treated with FM4-64 for 30 min. Images corresponding to 50 planes spaced by 0.6 μm through the cell volume were collected every 5 min using a spinning-disk confocal microscope (CSU-X1; Yookogawa). Maximum intensity projections are shown over time.
(AVI)

S12 Movie FM4-64 and RAB11A^Q70L^ apical trafficking in control cells. Control MDCK acini were transfected with RAB11A^Q70L^-GFP and treated with FM4-64 for 30 min. Images corresponding to 50 planes spaced by 0.6 μm through the cell volume were collected every 5 min using a spinning-disk confocal microscope (CSU-X1; Yookogawa). Maximum intensity projections are shown over time.
(AVI)

S13 Movie RAB11A^S22N^ expression alters FM4-64 apical trafficking in control cells. Control MDCK acini were transfected with RAB11A^S22N^-GFP and treated with FM4-64 for 30 min. Images corresponding to 50 planes spaced by 0.6 μm through the cell volume were collected every 5 min using a spinning-disk confocal microscope (CSU-X1; Yookogawa). Maximum intensity projections are shown over time.
(AVI)

S14 Movie RAB11A^WT^ does not rescue loss of HTT-induced defect in FM4-64 apical trafficking. shHTT2 MDCK acini were transfected with RAB11A^WT^-GFP and treated with FM4-64 for 30 min. Images corresponding to 50 planes spaced by 0.6 μm through the cell volume were collected every 5 min using a spinning-disk confocal microscope (CSU-X1; Yookogawa). Maximum intensity projections are shown over time.
(AVI)

S15 Movie RAB11A^Q70L^ rescues loss of HTT-induced defect in FM4-64 apical trafficking. shHTT2 MDCK acini were transfected with RAB11A^Q70L^-GFP and treated with FM4-64 for 30 min. Images corresponding to 50 planes spaced by 0.6 μm through the cell volume were collected every 5 min using a spinning-disk confocal microscope (CSU-X1; Yookogawa). Maximum intensity projections are shown over time.
(AVI)

S16 Movie RAB11A^S22N^ does not rescue loss of HTT-induced defect in FM4-64 apical trafficking. shHTT2 MDCK acini were transfected with RAB11A^S22N^-GFP and treated with FM4-64 for 30 min. Images corresponding to 50 planes spaced by 0.6 μm through the cell volume were collected every 5 min using a spinning-disk confocal microscope (CSU-X1; Yookogawa). Maximum intensity projections are shown over time.
(AVI)

## Abbreviations

AMIS: apical membrane initiation site
aPKC: atypical protein kinase C
BC: basal cell
CDC42: cell division control protein 42
CRB: crumbs
DLG: disc-large homolog
GAPs: GTPase-activating proteins
GEF: guanine nucleotide exchange factor
GFP: green fluorescent protein
HAP1: huntingtin-associated protein 1
HTT: huntingtin
KIF5: kinesin 1
LC: luminal cell
LGL: lethal giant larvae homolog
LGN: G protein regulator leucine-glycine-asparagine repeat protein
MDCK: Madin-Darby canine kidney
MMTV: Mouse mammary tumor virus
NUMA: nuclear mitotic apparatus protein
PAP: pre-apical patch
PATJ: PALS1-associated tight junction protein
PCX: podocalyxin
q-PCR: quantitative PCR
RAB11A: Ras-related protein 11A
RIP11/FIP5: RAB11-interacting protein
RT-PCR: reverse transcription PCR
shRNA: small hairpin RNA
STAT5A: signal transducer and activator of transcription 5A
TEBs: terminal end buds
WAP: whey acid protein
