# Supplementary material for: Huntingtin Is Required for Epithelial Polarity through RAB11A-Mediated Apical Trafficking of PAR3-aPKC
Source: PLoS Biol. 2015 May 5;13(5):e1002142. doi: 10.1371/journal.pbio.1002142 (PMC4420272; doi:10.1371/journal.pbio.1002142)
Supplement: S1 Data — (DOC) [file pbio.1002142.s001.doc]

**Statistical Analyses**

**Fig. 1A**

Data are from three independent cell sorting experiments: control: 5 mice per experiment, mutant: 5 mice per experiment

T test ***p<0.01

**Fig. 1C**

Data are from: control: 5 mice; mutant: 7 mice

T test *p<0.05; **p<0.01; ***p<0.001

**Fig. 1D**

Data are from: control: 5 mice; mutant: 7 mice

T test *p<0.05; **p<0.01; ***p<0.001

**Fig. 1E**

Data are from: control: 5 mice; mutant: 7 mice

T test ***p<0.001

**Fig. 1H**

Data are from: control: 5 mice; mutant: 7 mice

T test **p<0.01; ***p<0.001

**Fig. 1J**

Data are from: control: 5 mice; mutant: 7 mice

T test **p<0.01

**Fig. 2B**

Data are from: control: 6 mice; mutant: 6 mice

T test **p<0.01; ***p<0.001

**Fig. 2D**

Data are from: control: 6 mice; mutant: 6 mice

T test ***p<0.001

**Fig. 2E**

Data are from three independent experiments: control: 3 mice per experiment, mutant: 3 mice per experiment

T test ***p<0.01

**Fig. 2F**

Data are from three independent experiments: control: 3 mice per experiment, mutant: 3 mice per experiment

T test ***p<0.01

**Fig. 2G**

Data are from 2 independent experiments: control: 3 mice per experiment, mutant: 3 mice per experiment

T test ***p<0.01

**Fig. 3C**

Data are from: control: 4 mice; mutant: 4 mice

T test ***p<0.001

**Fig. 4C**

Data are from 2 independent experiments

ANOVA F[3,4] =11.189; p***<0.001

Fisher’s PLSD:

p-value

Control, shHTT1 0<0.001

Control, shHTT2 0<0.001

Control, si-HTT2+HTTFL 0<0.05

shHTT1, si-HTT2 0.1769

shHTT1+ si-HTT2+HTTFL 0<0.001

shHTT2+ si-HTT2+HTTFL 0<0.001

**Fig. 4I**

Data are from three independent experiments: Control: 125 acini, Control + PAR3: 102 acini, shHTT1: 149 acini, shHTT2: 114 acini, shHTT2 + HTT: 163 acini, shHTT2 + PAR3: 89 acini

ANOVA F[5,736] =35.012; p***<0.001

Fisher’s PLSD:

p-value

Control, Control+PAR3 0.4213

Control, shHTT1 0<0.001

Control, shHTT2 0<0.001

Control, si-HTT2+HTTFL 0.3617

Control, si-HTT2+PAR3 0<0.001

Control+PAR3, shHTT1 0<0.001

Control+PAR3, shHTT2 0<0.001

Control+PAR3, si-HTT2+HTTFL 0.1172

Control+PAR3, si-HTT2+PAR3 0<0.001

shHTT1, shHTT2 0.4981

shHTT1, si-HTT2+HTTFL 0<0.01

shHTT1, si-HTT2+PAR3 0.3649

shHTT2, si-HTT2+HTTFL 0<0.01

shHTT2, si-HTT2+PAR3 0.4778

shHTT2+HTTFL, si-HTT2+PAR3 0<0.001

**Fig. 4J**

Data are from three independent experiments: Control: 125 acini, Control + PAR3: 102 acini, shHTT1: 149 acini, shHTT2: 114 acini, shHTT2 + HTT: 163 acini, shHTT2 + PAR3: 89 acini

ANOVA F[5,736] =35.012; p***<0.001

Fisher’s PLSD:

p-value

Control, Control+PAR3 0.3774

Control, shHTT1 0<0.001

Control, shHTT2 0<0.001

Control, si-HTT2+HTTFL 0.2951

Control, si-HTT2+PAR3 0<0.001

Control+PAR3, shHTT1 0<0.001

Control+PAR3, shHTT2 0<0.001

Control+PAR3, si-HTT2+HTTFL 0.2659

Control+PAR3, si-HTT2+PAR3 0<0.001

shHTT1, shHTT2 0.2782

shHTT1, si-HTT2+HTTFL 0<0.001

shHTT1, si-HTT2+PAR3 0.1247

shHTT2, si-HTT2+HTTFL 0<0.001

shHTT2, si-HTT2+PAR3 0.3094

shHTT2+HTTFL, si-HTT2+PAR3 0<0.001

**Fig. 5F**

Data are from three independent experiments: Control: 94 acini, Noco 10 µM 90 min: 67 acini, Noco 5 µM 16h: 72 acini

ANOVA F[2,230] =8.227; p***<0.001

Fisher’s PLSD:

p-value

Control, Noco 10 µM 90 min 0.3852

Control, Noco 5 µM 16h <0.001

Noco 10 µM 90 min, Noco 5 µM 16h <0.001

**Fig. 5H**

Data are from two independent experiments: Control: 22 24h acini and 26 d4 acini, shHTT1: 25 24h acini and 25 d4 acini

T test ***p<0.001

**Fig. 5K**

Data are from two independent experiments: si-Control: 30 acini, si-kinesin 1: 28 acini

T test ***p<0.001

**Fig. 6G**

Data are from three independent experiments: Control+RAB11A^WT^: 59 acini, Control+RAB11A^Q70L^: 54 acini, Control+RAB11A^S22N^: 66 acini, shHTT2+RAB11A^WT^: 60 acini, shHTT2+RAB11A^Q70L^: 72 acini, shHTT2+RAB11A^S22N^: 93 acini

ANOVA F[5,398] =24.312; p***<0.001

Fisher’s PLSD:

p-value

Control+RAB11A^WT^, Control+RAB11A^Q70L^ 0.2964

Control+RAB11A^WT^, Control+RAB11A^S22N^ 0<0.001

Control+RAB11A^WT^, shHTT2+RAB11A^WT^ 0<0.001

Control+RAB11A^WT^, shHTT2+RAB11A^Q70L^ 0.3209

Control+RAB11A^WT^, shHTT2+RAB11A^S22N^ 0<0.001

Control+RAB11A^Q70L^, Control+RAB11A^S22N^ 0<0.001

Control+RAB11A^Q70L^, shHTT2+RAB11A^WT^ 0<0.001

Control+RAB11A^Q70L^, shHTT2+RAB11A^Q70L^ 0.1094

Control+RAB11A^Q70L^, shHTT2+RAB11A^S22N^ 0<0.001

Control+RAB11A^S22N^, shHTT2+RAB11A^WT^ 0<0.05

Control+RAB11A^S22N^, shHTT2+RAB11A^Q70L^ 0<0.001

Control+RAB11A^S22N^, shHTT2+RAB11A^S22N^ 0.4782

shHTT2+RAB11A^WT^, shHTT2+RAB11A^Q70L^ 0<0.001

shHTT2+RAB11A^WT^, shHTT2+RAB11A^S22N^ 0.3965

shHTT2+RAB11A^Q70L^, shHTT2+RAB11A^S22N^ 0<0.001

**Fig. 6H**

Data are from three independent experiments: Control+RAB11A^WT^: 59 acini, Control+RAB11A^Q70L^: 54 acini, Control+RAB11A^S22N^: 66 acini, shHTT2+RAB11A^WT^: 60 acini, shHTT2+RAB11A^Q70L^: 72 acini, shHTT2+RAB11A^S22N^: 93 acini

ANOVA F[5,398] =21.185; p***<0.001

Fisher’s PLSD:

p-value

Control+RAB11A^WT^, Control+RAB11A^Q70L^ 0.3549

Control+RAB11A^WT^, Control+RAB11A^S22N^ 0<0.001

Control+RAB11A^WT^, shHTT2+RAB11A^WT^ 0<0.001

Control+RAB11A^WT^, shHTT2+RAB11A^Q70L^ 0.4521

Control+RAB11A^WT^, shHTT2+RAB11A^S22N^ 0<0.001

Control+RAB11A^Q70L^, Control+RAB11A^S22N^ 0<0.001

Control+RAB11A^Q70L^, shHTT2+RAB11A^WT^ 0<0.001

Control+RAB11A^Q70L^, shHTT2+RAB11A^Q70L^ 0.2654

Control+RAB11A^Q70L^, shHTT2+RAB11A^S22N^ 0<0.001

Control+RAB11A^S22N^, shHTT2+RAB11A^WT^ 0<0.05

Control+RAB11A^S22N^, shHTT2+RAB11A^Q70L^ 0<0.001

Control+RAB11A^S22N^, shHTT2+RAB11A^S22N^ 0.1836

shHTT2+RAB11A^WT^, shHTT2+RAB11A^Q70L^ 0<0.001

shHTT2+RAB11A^WT^, shHTT2+RAB11A^S22N^ 0.1924

shHTT2+RAB11A^Q70L^, shHTT2+RAB11A^S22N^ 0<0.001

**S1B Fig.**

Data are from: control: 4 mice; mutant: 3 mice

T test p=0.1854

**S1C Fig.**

Data are from three independent cell sorting experiments: control: 3 mice per experiment, mutant: 3 mice per experiment

T test p=0.3691

**S2C Fig.**

Day 8: Data are from three independent experiments: shControl: 47 acini, shHTT: 32 acini

Day 10: Data are from three independent experiments: shControl: 45 acini, shHTT: 30 acini

Day 20: Data are from three independent experiments: shControl: 31 acini, shHTT: 30 acini

T test ***p<0.001

**S2E Fig.**

Day 10: Data are from three independent experiments: shControl: 34 acini, shHTT: 35 acini

Day 20: Data are from three independent experiments: shControl: 38 acini, shHTT: 46 acini

T test ***p<0.001

**S2F Fig.**

Day 10: Data are from three independent experiments: shControl: 35 acini, shHTT: 35 acini

Day 20: Data are from three independent experiments: shControl: 38 acini, shHTT: 30 acini

T test **p<0.01

**S3C Fig.**

Data are from: control: 3 mice; mutant: 3 mice

T test ***p<0.001

**S3E Fig.**

Data are from three independent experiments: shControl: 56 acini, shHTT: 67 acini

T test ***p<0.001

**S3F Fig.**

Data are from three independent experiments: shControl: 56 acini, shHTT: 67 acini

T test ***p<0.001
