# Supplementary figures and images for: Huntingtin Is Required for Epithelial Polarity through RAB11A-Mediated Apical Trafficking of PAR3-aPKC
Source: PLoS Biol. 2015 May 5;13(5):e1002142. doi: 10.1371/journal.pbio.1002142 (PMC4420272; doi:10.1371/journal.pbio.1002142)

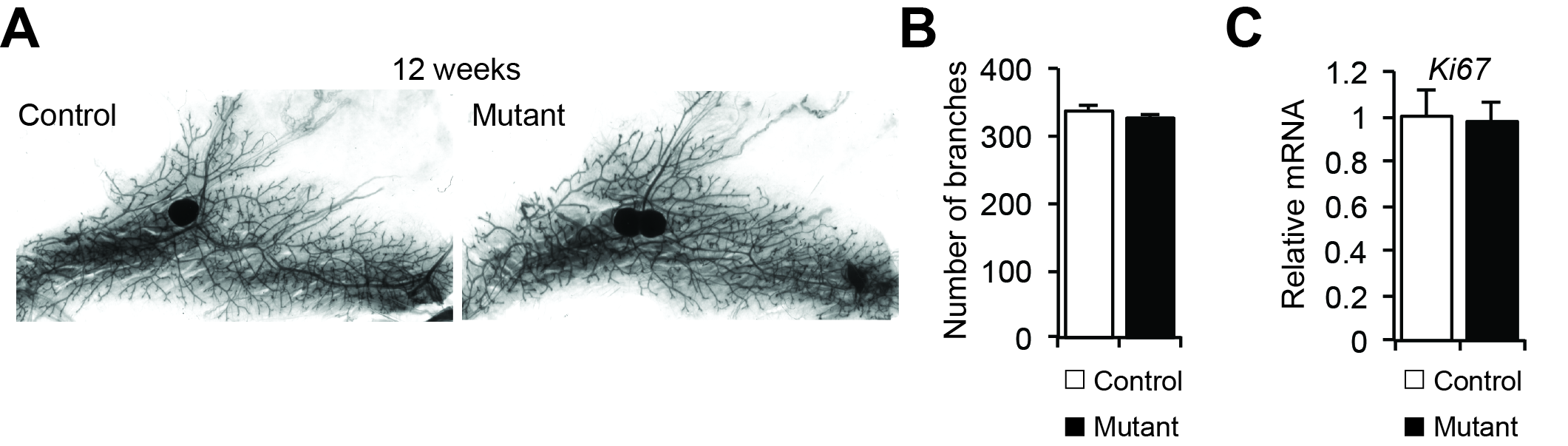

Supplement: S1 Fig — (A) Carmine-stained whole mounts of 12-wk-old virgin mammary glands. (B) Number of branches in 12-wk-old virgin mammary glands. (C) Quantitative real-time RT-PCR analysis of Ki67 gene in mammary epithelial cells from 12-wk-old virgin mice. Data are presented as means obtained in three independent experiments (control: three mice per experiment, mutant: three mice per experiment). Error bars, SEM. (TIF) [file pbio.1002142.s002.tif]

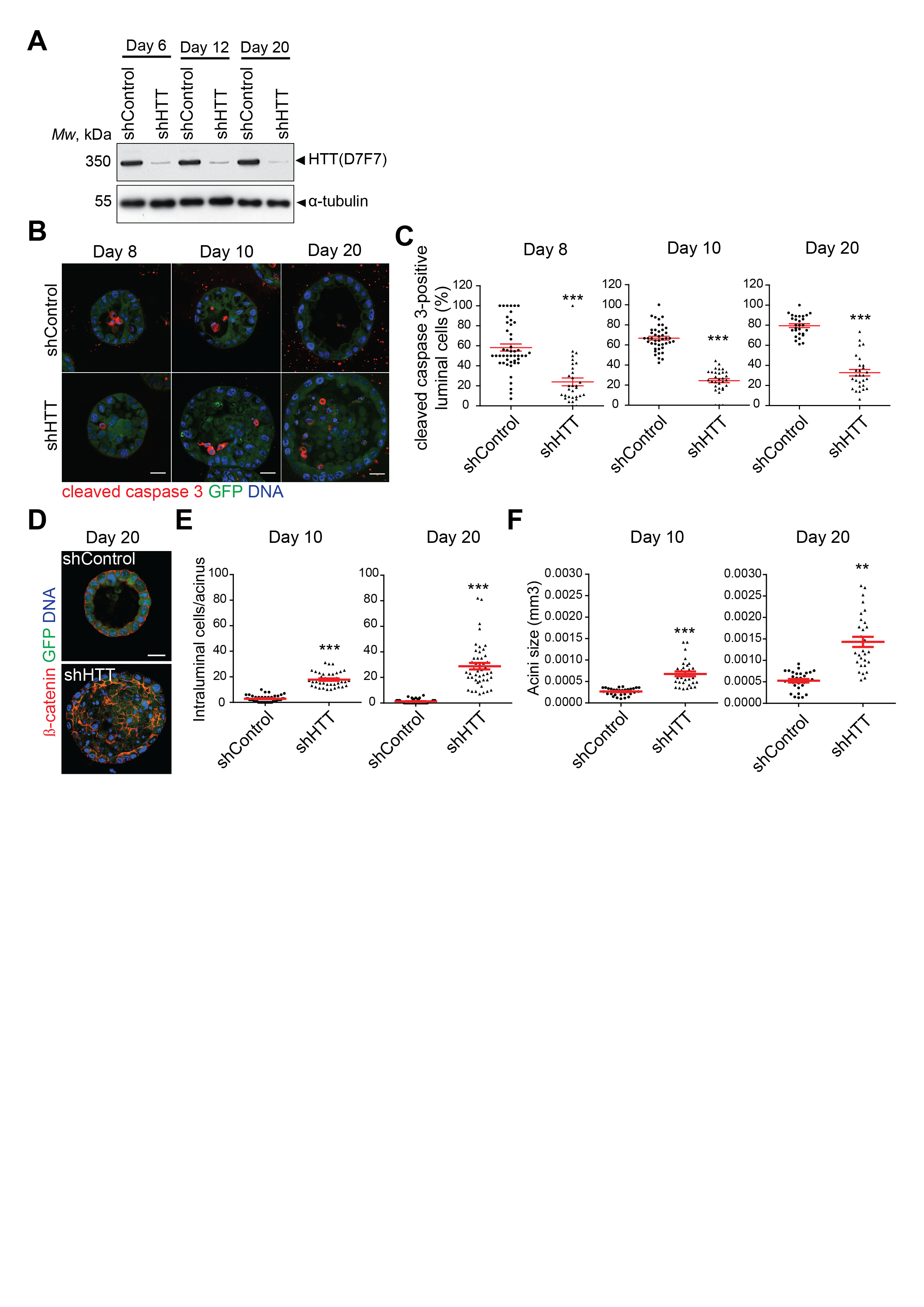

Supplement: S2 Fig — (A) Western blotting of extracts from day 6, 12, and 20 shControl and shHTT MCF-10A cells in 3-D culture. (B) Day 8, 10, and 20 shControl and shHTT MCF-10A 3-D acini stained for cleaved caspase 3. Scale bar, 10 μm. (C) Percentage of cleaved caspase 3-positive luminal cells: Day 8 (shControl: n = 47 acini, shHTT: n = 32 acini); Day 10 (shControl: n = 45 acini, shHTT: n = 30 acini); Day 20 (shControl: n = 31 acini, shHTT: n = 30 acini). (D) Day 20 shControl and shHTT MCF-10A acini stained for ß-catenin. Scale bar, 10 μm. (E) Quantification of the number of intraluminal cells in day 10 and 20 shControl and shHTT MCF-10A acini: Day 10 (shControl: n = 34 acini, shHTT: n = 35 acini); Day 20 (shControl: n = 38 acini, shHTT: n = 46 acini). (F) Quantification of day 10 and 20 shControl and shHTT MCF-10A acini size: Day 10 (shControl: n = 35 acini, shHTT: n = 35 acini); Day 20 (shControl: n = 38 acini, shHTT: n = 30 acini). Error bars, SEM. ** p<0.01; *** p<0.001. (TIF) [file pbio.1002142.s003.tif]

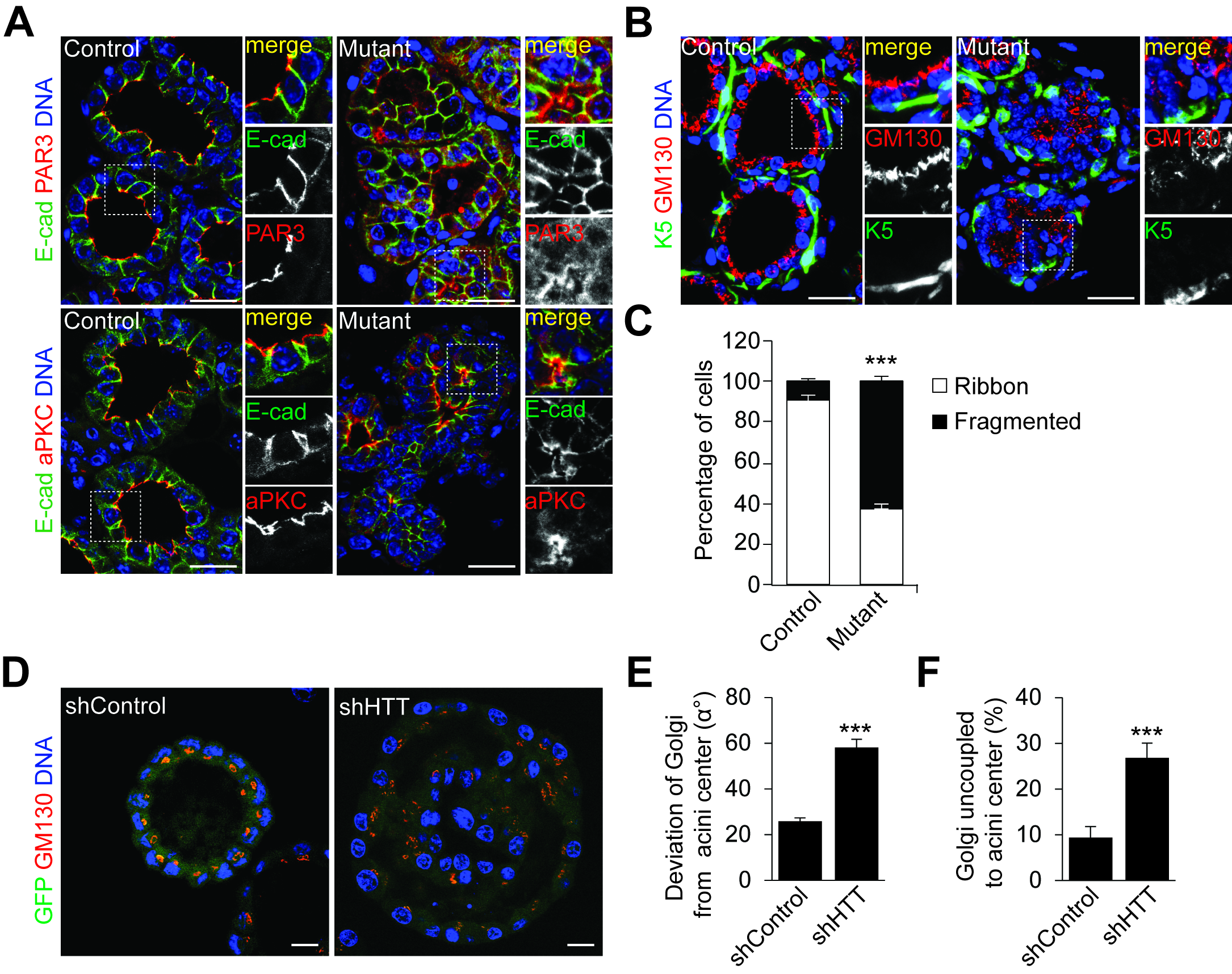

Supplement: S3 Fig — (A) Mammary gland sections stained for E-cadherin and PAR3 or aPKC. (B) Mammary gland sections stained for keratin 5 (K5) and GM130. (C) Percentage of LCs showing ribbon-like and fragmented GM130 (control: n = 3 mice; mutant: n = 3 mice). (D) Day 20 shControl and shHTT MCF-10A 3-D acini stained for GM130. (E) Deviation of the Golgi from acini center (α°) (shControl: n = 56 acini, shHTT: n = 67 acini). (F) Percentage of acini with Golgi uncoupled to center (shControl: n = 56 acini, shHTT: n = 67 acini). All scale bars, 10 μm; Error bars, SEM; ***p<0.001. (TIF) [file pbio.1002142.s004.tif]

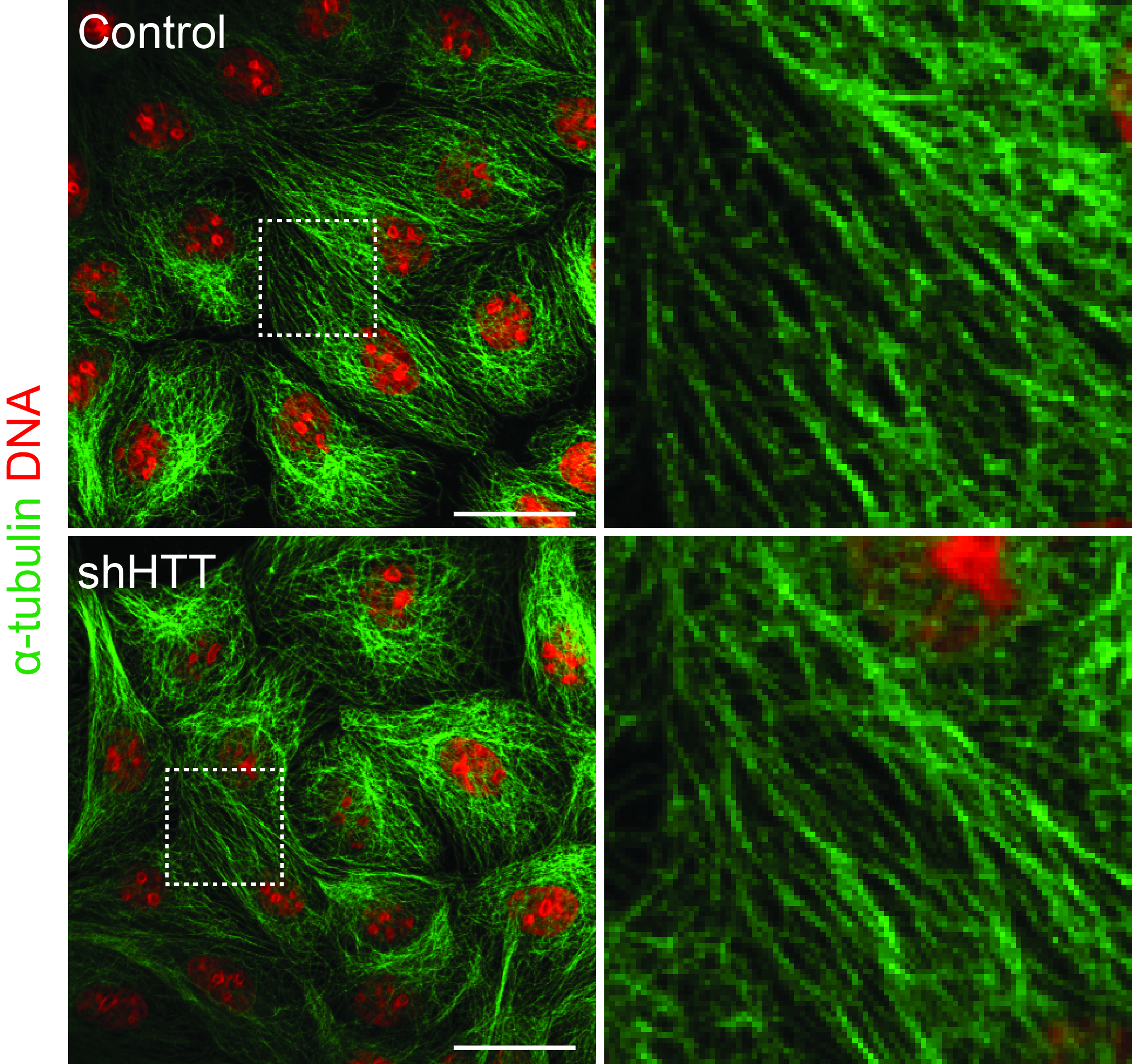

Supplement: S4 Fig — MCF10-10A cells stained for α-tubulin. Scale bar, 10 μm. (TIF) [file pbio.1002142.s005.tif]

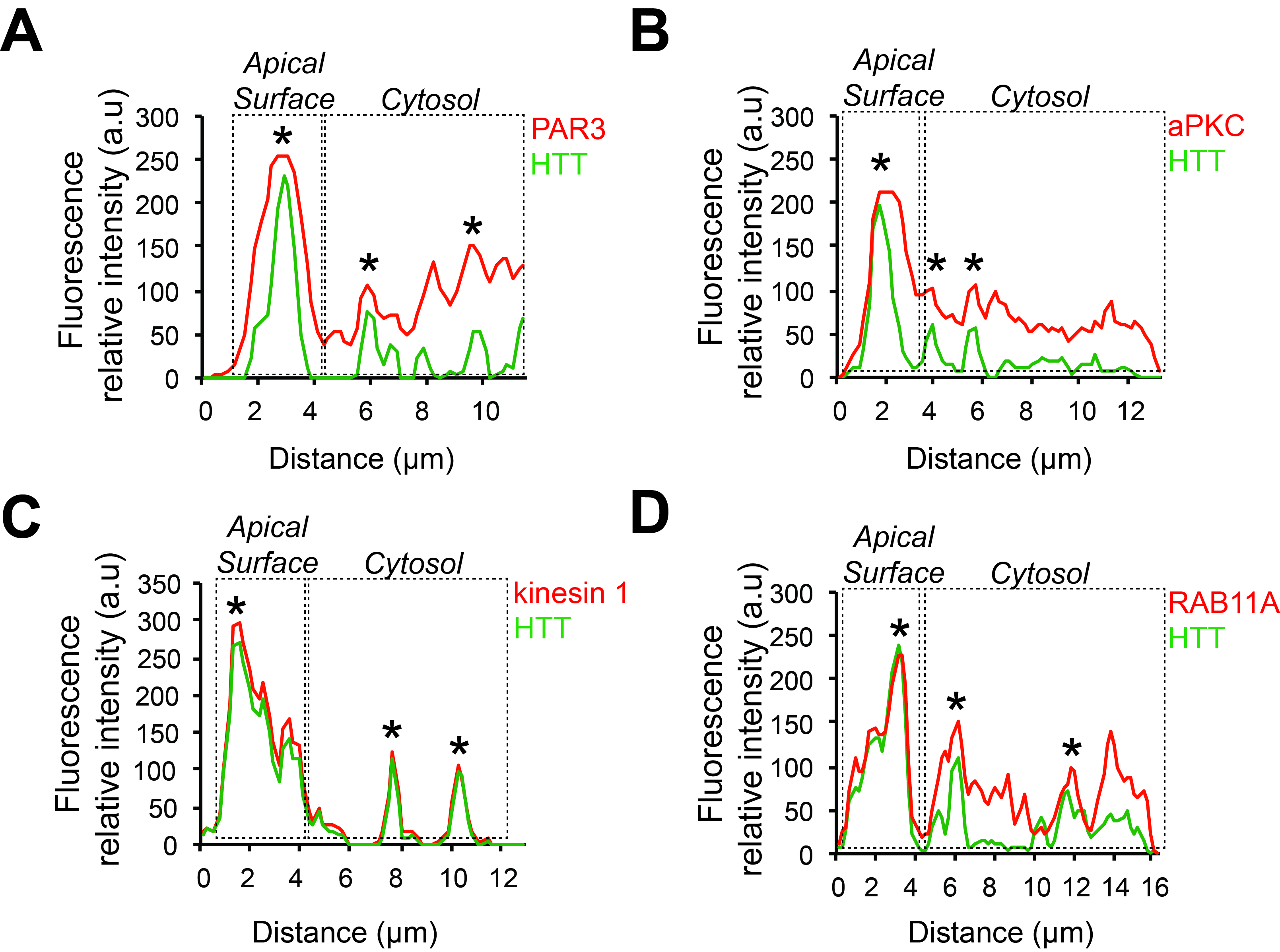

Supplement: S5 Fig — Representative line-scan analysis of overlap and non-overlap of HTT with PAR3 (A), aPKC (B), kinesin 1 (C) and RAB11A (D) (relative fluorescence intensity; at least 20 cells were analyzed per condition). Asterisks indicate colocalizations. (TIF) [file pbio.1002142.s006.tif]

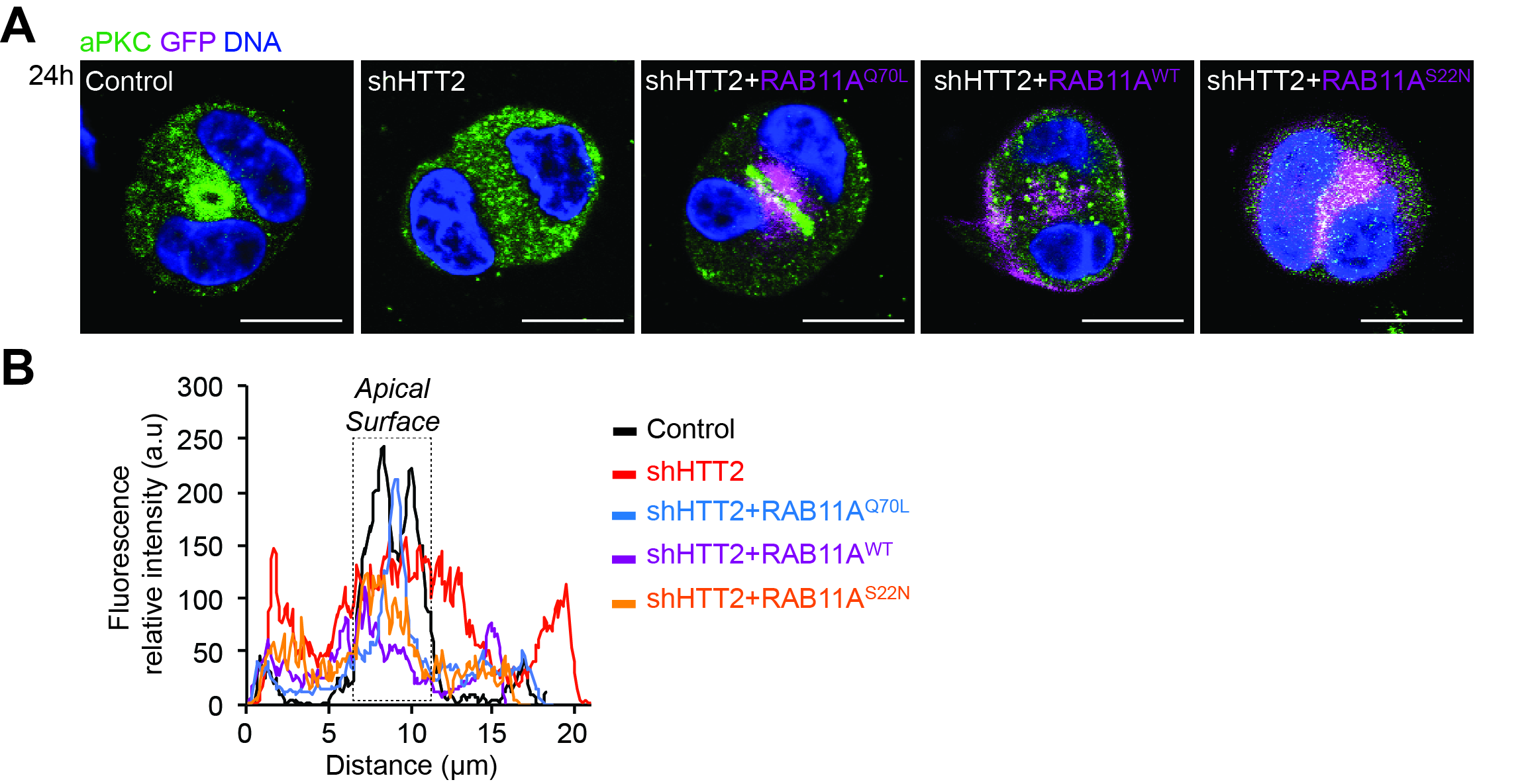

Supplement: S6 Fig — (A) Twenty-four–hour MDCK 3-D cultures transfected with RAB11AQ70L, RAB11AWT, or RAB11AS22N, stained for aPKC. RAB11A is tagged with GFP, and fluorescence is displayed in magenta, and the colocalization of aPKC and RAB11A appears in white. (B) Representative line-scan analysis (relative fluorescence intensity; at least 20 cells were analyzed per condition). (TIF) [file pbio.1002142.s007.tif]
